# Exposure duration shapes GenX-induced transcriptomics responses in 3D HepG2/C3A spheroids

**DOI:** 10.3389/ftox.2026.1966541

**Published:** 2026-10-14

**Authors:** Chanhee Kim, Abderrahmane Tagmount, Zhaohan Zhu, Benjamin Aiken, W. Brad Barbazuk, Rhonda Bacher, Christopher D. Vulpe

**Affiliations:** 1 Department of Physiological Sciences, Center for Human and Environmental Toxicology, College of Veterinary Medicine, University of Florida, Gainesville, FL, United States; 2 Department of Biostatistics, University of Florida, Gainesville, FL, United States; 3 Department of Biology, University of Florida, Gainesville, FL, United States; 4 University of Florida Genetics Institute, University of Florida, Gainesville, FL, United States

**Keywords:** 3D spheroid, GenX, HepG2/C3a, low-dose, non-monotonic DEG trend, time-dependent

## Abstract

Hexafluoropropylene oxide dimer acid (GenX), a replacement for legacy per- and polyfluoroalkyl substances (PFAS), is increasingly detected in the environment, yet its long-term toxicity remains poorly characterized. Current safety assessments rely largely on short-term, high-dose studies that may not capture the biological consequences of longer-term, low-dose exposure. To address this gap, we employed monocellular 3D HepG2/C3A hepatic spheroids cultured in a continuously rotating bioreactor system (ClinoStar) to systematically evaluate dose- and time-dependent mRNA changes in response to GenX under environmentally relevant conditions. Spheroids were exposed to GenX (0.08–50 μM, spanning environmentally relevant to mechanistically informative concentrations) for acute (4 days) and long-term (4 weeks) durations, followed by genome-wide TempO-Seq transcriptomic profiling and benchmark dose (BMD) modeling. Following actue exposure, the greatest number of differentially expressed genes (DEGs) was observed at 0.4 μM, with fewer DEGs at higher concentrations. Long-term exposure produced a different concentration-dependent distribution of DEG with the greatest number observed at 50 μM. Notably, acute and long-term exposures elicited qualitatively distinct mRNA changes with low concordance across matched concentrations, demonstrating that exposure duration was a major determinant of mRNA changes. Acute low-dose GenX exposure preferentially modulated mRNA encoding components of cell cycle-related pathways, whereas acute higher dose exposures suppress mRNA levels of the transcripts encoding constituents of lipid metabolic pathways and increase expression of mRNA encoding proteins involved in stress- and toxicity-associated signaling. Long-term exposure revealed a different pattern of changes in mRNA expression not observed under acute exposure conditions, including suppression of mRNA expression levels of transcripts encoding cellular components involved in lipid-related pathways at the lowest concentration tested. At higher concentrations, mRNA levels encoding transcritps of components of multiple metabolic pathways were altered. Benchmark dose modeling identified a significantly lower transcriptomic point of departure (tPOD) for long-term exposure as compared to acute exposure, suggesting increased cellular response to prolonged GenX exposure and supporting the relevance of long-term models for human exposure assessment. Collectively, these findings demonstrate that GenX elicits distinct transcriptomic responses in HepG2/C3A spheroids depending on both concentration and exposure duration. This study, therefore, highlights the importance of incorporating long-term 3D hepatic *in vitro* models and transcriptomic endpoints into PFAS risk assessment and suggests that conventional short-term assays may underestimate the biological impact of sustained low-dose exposure. Key message (Impact of the study): This study provides systematic comparisons of short term (4 days) versus longer term (4 weeks), environmentally relevant GenX exposure in monocellular HepG2/C3A hepatic spheroids, revealing non-monotonic, time-dependent changes in mRNA levels encoding cellular components of lipid metabolism-related pathways with potential implications for appropriate dose and time exposure parameters for use in New Approach Methods to be applied in risk assessment.

## 1 Introduction

Perfluoroalkyl substances (PFAS) alternatives have been developed to replace ‘legacy’ PFAS that exhibit extreme environmental persistence and bioaccumulation in humans and wildlife due to their resistance to degradation (Cheng et al., 2025). Among these alternatives, hexafluoropropylene oxide dimer acid (HFPO-DA; GenX) was introduced as a replacement for perfluorooctanoic acid (PFOA) following increasing regulatory restrictions on legacy PFAS (Guo et al., 2025; Wen et al., 2020). However, growing evidence indicates that GenX is also an emerging environmental contaminant of concern (Blake et al., 2020; Hopkins et al., 2018). Reflecting these concerns, the U.S. Environmental Protection Agency (EPA) released a human health toxicity assessment for GenX in 2021 and subsequently included GenX among PFAS subject to regulatory monitoring and drinking water standards (US EPA, 2021). More recently, the U.S. EPA announced the final drinking water regulation for six PFAS, including GenX, pointing out the urgent human health concern posed by this chemical (US EPA, 2022). GenX has been detected in drinking water, groundwater, surface water, soil, and vegetation near fluorochemical manufacturing facilities, with concentrations in some contaminated regions exceeding recommended safety limits (Brandsma et al., 2019; Gebbink et al., 2017). Despite increasing environmental detection, evidence of human exposure, and growing regulatory attention, the long-term health effects and human-relevant mechanisms of GenX toxicity remain insufficiently characterized (Guo et al., 2025; Kancharla et al., 2022; Pan et al., 2018).

Accumulating epidemiological and experimental studies have identified potential adverse effects of GenX (Beale et al., 2022; Conley et al., 2019), including GenX-induced toxicity in the liver (Ren et al., 2024; Yoo et al., 2023), reproductive system (Peng et al., 2022; Zhang et al., 2025), endocrine systems (Coperchini et al., 2020), development (Davidsen et al., 2021), and immune function (Robarts et al., 2022). The liver is the primary site of GenX accumulation and one of its most sensitive toxicity targets (Guo et al., 2022; Wan et al., 2024). Hepatic effects associated with GenX exposure include metabolic disruption, liver enlargement (i.e., hepatomegaly), altered lipid homeostasis, and extensive transcriptional reprogramming (Teschke, 2019). Transcriptomic and systems toxicology studies in rodents, fish, and *in vitro* models have demonstrated that GenX alters pathways involved in lipid metabolism, bile acid homeostasis, immune signaling, and cellular stress responses (Chappell et al., 2020; Blake et al., 2022; Heintz et al., 2022; Rericha et al., 2024; Zang et al., 2025). Further, a recent study reported that while most PFAS suppress cholesterol biosynthesis and innate immunity, GenX exposure did not suppress cholesterol biogenesis genes and instead upregulated innate immune signaling genes in human liver spheroids (Addicks et al., 2025). Dysregulation of bile acid metabolism has also emerged as a potential contributor to GenX-induced hepatotoxicity, with *in vivo* studies demonstrating increased total bile acids, elevated primary bile acids, and reduced secondary bile acids following exposure (Ren et al., 2025; Zhang et al., 2025b). Collectively, these findings indicate that GenX toxicity likely involves multiple interacting pathways and that a unified human-relevant mechanistic framework remains to be developed.

Importantly, most current knowledge of GenX toxicity is derived from acute or relatively short-term exposure studies conducted at moderate to high concentrations, particularly in the case of *in vitro* approaches (Wen et al., 2020; Yoo et al., 2021). Such approaches may not adequately capture the biological consequences of prolonged low-dose exposure that more closely reflect real-world human exposure scenarios. Another limitation of previous studies is the limited use of *in vitro* hepatic models that can support prolonged culture while retaining key liver-like features and adaptive responses to long-term chemical exposure. Conventional two-dimensional (2D) hepatocyte cultures often fail to recapitulate the cellular architecture, metabolic competence, and long-term functionality of human liver tissue (Godoy et al., 2013; Kapałczyńska et al., 2018; Kim et al., 2026). Compared with conventional 2D cultures, 3D hepatic models, including monocellular spheroids derived from HepG2, HepaRG, or primary hepatocytes and more complex iPSC-derived spheroids or organoids, can better retain selected hepatic phenotypes and support prolonged culture (Kim et al., 2026; Kim et al., 2024). However, their biological complexity varies substantially, and monocellular spheroids do not reproduce the multicellular architecture or intercellular signaling of intact human liver. Incorporation of environmentally relevant exposure concentrations further enhances the utility of these systems for human health risk assessment.

Most experimental studies investigating GenX toxicity, particularly those using human *in vitro* models, relied on relatively short exposure periods ranging from several hours to only a few days. For example, recent mechanistic studies examining GenX toxicity in human HepG2 cells and rat thyroid FRTL-5 cells employed exposure durations of up to 72 h (Coperchini et al., 2020; Yoo et al., 2021). Similarly, a recent transcriptomic study evaluating 24 different PFAS, including GenX, in human primary hepatocyte spheroids examined responses after 24 h and 10 days of exposure (Addicks et al., 2025). While these studies have provided valuable insights into the early molecular events and mechanisms underlying GenX toxicity, it remains unclear whether such relatively short-term exposure paradigms adequately capture the biological responses associated with sustained or long-term exposure.

Although short-term exposure studies are useful for identifying early molecular events and mechanisms of action, their relevance to sustained human exposure remains uncertain because transcriptional responses may evolve through adaptation, cumulative perturbation, or transition to distinct biological states during long-term exposure. This issue is particularly important for quantitative transcriptomics, in which benchmark dose or concentration modeling is increasingly used to derive transcriptomic points of departure (tPODs) for chemical hazard and risk assessment. Short-term tPODs have shown encouraging concordance with long-term apical effects, which allowed them to be developed as efficient tools for screening and regulatory assessment (Pagé-Larivière et al., 2019). However, tPOD estimates can vary with exposure duration in a chemical-dependent manner, and there remains no universal basis for assuming that a tPOD derived from an acute *in vitro* exposure will adequately represent the potency or biological processes associated with long-term exposure (Carpi et al., 2024). Direct comparison of acute and long-term exposure conditions is therefore needed to determine whether short-term transcriptomic responses provide a sufficiently protective and biologically representative basis for assessing chemicals such as GenX for which there is sustained human exposure.

In the present study, we systematically characterized dose- and time-dependent mRNA changes in response to GenX using a monocellular 3D HepG2/C3A hepatic spheroid model cultured in a rotating bioreactor system. To determine how exposure duration shapes biological responses, spheroids were exposed to GenX across environmentally relevant and mechanistically informative concentrations under acute (4 days) and long-term (4 weeks) exposure conditions. We hypothesized that prolonged exposure would alter both the magnitude and nature of GenX-induced mRNA changes, revealing biological effects that may not be predicted from conventional short-term studies. Through transcriptomic profiling and benchmark dose modeling, this study provides insight into the mechanisms and molecular response to long-term GenX exposure and highlights the importance of exposure duration in interpreting transcriptomic data for PFAS risk assessment.

## 2 Methods

### 2.1 HepG2/C3A 3D spheroid culture, GenX exposure, and cell viability assay

HepG2/C3A cells (ATCC) were maintained in Minimum Essential Medium (MEM, Gibco) supplemented with 10% fetal bovine serum (FBS), 1% non-essential amino acids (Gibco), and 1% Antibiotic-Antimycotic (Gibco) under standard culture conditions (37 °C, 5% CO_2_). Three-dimensional (3D) spheroids were generated using the ClinoStar rotating bioreactor system (CelVivo) as previously described (Kim et al., 2026; Kim et al., 2024). The resulting spheroids were monocellular and contained only HepG2/C3A cells. Briefly, HepG2/C3A cells were seeded onto a Spherical Plate 5D (SP5D) plate at 500 cells per microwell and allowed to aggregate for 24 h at 37 °C and 5% CO_2_. The pre-aggregated spheroids were subsequently transferred into ClinoReactors and maintained under continuous rotation in the ClinoStar system. Culture medium was replaced two to three times per week throughout the experiment. To evaluate concentration- and duration-dependent transcriptomic responses, mature (a day after transfer of pre-aggregates) HepG2/C3A spheroids were exposed to hexafluoropropylene oxide dimer acid (HFPO-DA; GenX) at nominal concentrations of 0.08, 0.4, 2, 10, or 50 μM. Vehicle-treated (dimethyl sulfoxide, DMSO) spheroids served as controls. Two exposure durations were evaluated: acute exposure (4 days) and long-term exposure (4 weeks). The acute exposure consisted of a single administration of GenX at the beginning of the exposure period, without medium replacement. For long-term studies, freshly prepared exposure medium containing GenX was replenished during routine medium changes every 3–4 days throughout the exposure period. At the end of each exposure period, spheroids were harvested for transcriptomic analysis. Three independent biological replicates were collected for each treatment group. Cell viability was additionally assessed in HepG2/C3A cells following 96 h of GenX exposure using a luminescence-based assay (CellTiter-Glo® 3D Cell Viability Assay, Promega) following the previously established protocol (Kim et al., 2024). Luminescence signals were normalized to those of the corresponding vehicle-treated controls, and cell viability was expressed as a percentage of control.

### 2.2 TempO-Seq

Transcriptome profiling was performed using the TempO-Seq Human Whole Transcriptome Assay panel (BioSpyder Technologies Inc., Carlsbad, California) as previously described (Kim et al., 2024). Briefly, total RNA was extracted and purified from each sample (spheroids) using a RNeasy Plus Mini Kit (Qiagen) following the manufacturer’s instructions. Throughout the RNA extraction procedure, we measured the 260/280 and 260/230 absorbance ratios to determine RNA purity and the presence of contaminants in our biological samples. Genomic DNA contamination was removed using DNase I (Ambion, UK) according to the manufacturer’s instructions. All sample O.D. values showed a 260/280 ratio of less than 1.8 and a 260/230 ratio of less than 2.0. The resultant RNA samples were then diluted with sterile water to obtain a consistent concentration of 100 ng/μL across all samples. An equal volume of 2X TempO-Seq Enhanced Lysis Buffer was added to each RNA sample, which provides an assay-ready sample in 1X TempO-Seq Enhanced Lysis Buffer. The assay-ready samples were annealed to the detector oligo (DO, targeting and binding a single RNA of interest) mix using the following steps: the assay-ready samples were incubated on plate at 70 °C for 10 min, then ramped to 45 °C for 50 min with the same ramp rate; the plate was maintained at 45 °C for 16 h, then cooled to 25 °C. Annealing was followed by nuclease digestion (37 °C for 1.5 h, the type of nuclease is not indicated by BioSpyder protocol) to remove excess oligos, and ligating (by incubating for 1 h at 37 °C, then immediately raised to 80 °C for 15 min) of a pair of DOs. The ligated product was PCR amplified using primers that contain sample tag (index) sequences and adaptors required for sequencing using the following. PCR cycles: 1. 37 °C for 10 min, 2. 95 °C for 1 min, 3. 25 cycles of 95 °C 10sec; 65 °C 30sec; 68 °C 30 s, 4. 68 °C for 60 s, 5. Hold at 25 °C. The amplified assay products are then pooled and purified/concentrated using the Macherey-Nagel NucleoSpin Gel and PCR Cleanup kit (Takara Bio USA, Inc., Mountain View, California). The library was sequenced using a NovaSeqX (performed at BioSpyder).

Primary data analysis was performed on a raw fastq files by BioSpyder using the TempO-SeqR (v.3.0) program. Initially, reads were extracted from the bcl files and demultiplexed with bcl2fastq v.2.20.0.42. The fastq files were processed with the TempO-SeqR (v.3.0), which uses star v.2.5 to align the reads, and the count function from QuasR to extract feature counts using a gtf reference. A raw gene_count table that details the signal levels (counts) for each probe in each sample was provided by BioSpyder. The raw gene_count matrix, which contained 19,683 unique genes across 36 samples, representing three replicates for each concentration and exposure duration. All expression entries were non-negative integers, with no missing values or duplicated gene identifiers. For this assessment, CPM values were calculated by dividing each gene count by the corresponding sample’s total gene count and multiplying by one million. Genes with at least 0.5 CPM in at least one of the 36 samples were retained. This criterion retained 14,365 genes and excluded 5,318 genes, including 195 genes with zero counts across all samples. Replicate consistency was assessed using pairwise Pearson correlations of log_2_(CPM + 1) values for the retained genes. Within-treatment correlations ranged from 0.9938 to 0.9970, indicating high concordance of global expression profiles among replicates. All 36 samples were included in this additional assessment. These checks address expression-matrix integrity and replicate consistency; they do not independently establish sequencing read quality or alignment performance.

### 2.3 Bioinformatics

All bioinformatics analyses for the raw gene_count data were conducted using integrated Differential Expression and Pathway analysis version 1.13 (iDEPv1.13) (Ge et al., 2018). Counts were count per million (CPM) normalized and genes without 0.5 CPM in at least one sample were removed from analysis. To facilitate clustering analyses and PCA, the filtered gene count data were log2 transformed using the log_2_ (cpm + c) function in the EdgeR package implemented in iDEPv1.13 (Ge et al., 2018). To assess sample variability, Pearson correlations of the log_2_ (cpm + c) values were plotted to visually assess within sample similarity and between sample variability.

#### 2.3.1 Differentially expressed genes

iDEPv1.13 was used to perform differential gene expression analysis on all pairs of sample groups using the DESeq2 (Love et al., 2014) R package implemented in iDEPv1.13. Expression was filtered at a fold-change > 2 and a false discovery rate (FDR) threshold of < 0.05.

#### 2.3.2 Biological pathway enrichment analysis

The resulting differentially expressed genes (DEGs) between comparison samples were then used to conduct enrichment analysis using Gene Ontology (Ensembl 92) and Kyoto Encyclopedia of Genes and Genomes database (KEGG, Release 86.1). The background genes used for the enrichment analysis are the filtered genes from the genome-wide original gene list. Enrichment p-values are calculated using a one-sided hypergeometric test, then adjusted for multiple testing with the Benjamini-Hochberg procedure and converted to FDR. The pathways are first filtered based on an FDR threshold of 0.05, and then the significant pathways are sorted by FDR. Functional analysis of DEGs was also conducted using STRING-DB_v12.0 (STRING: functional protein association networks, accessed 07/08/2026) for DEGs identified following 4-day and 4-week exposures to generate clustered networks. Upregulated and downregulated genes were analyzed separately. Gene symbols were mapped to *Homo sapiens* proteins, and only successfully mapped genes were included in the analysis. Functional enrichment was assessed using GO-BP annotations available in STRING, with the whole human genome used as the statistical background. Enriched GO-BP terms were ranked according to false discovery rate (FDR), and terms with an FDR < 0.05 were considered statistically significant. Enrichment results were visualized using −log_10_(FDR) on the x-axis, with point size indicating the number of DEGs associated with each pathway or GO term. Results obtained from upregulated and downregulated DEG sets were distinguished by color. Comparisons across exposure conditions considered enrichment significance, contributing DEG counts, and the biological identities of the enriched terms. These metrics were used to characterize enrichment patterns, rather than as direct measures of biological effect magnitude.

#### 2.3.3 Concentration-response trend analysis

Raw gene expression data were normalized using Median Normalization (Anders and Huber, 2010) to account for variation in library size and sequencing depth across samples. Briefly, this method first calculates a ‘size factor’ for each sample, accounting for variations in sequencing depth. Then the method divides each gene’s expression value by its corresponding size factor. The result is a set of normalized expression values that are adjusted to a common scale across all samples.

To investigate transcriptomic responses across increasing GenX concentrations, we adapted the Trendy package (Bacher et al., 2018), originally developed for time-course gene expression analysis, to identify genes exhibiting non-linear dose-response relationships. In this framework, GenX concentration was treated as an ordered variable analogous to time. Trendy fits a series of segmented regression models to each gene and identifies statistically significant breakpoints representing changes in the direction or magnitude of expression responses across the dose range. Separate analyses were conducted for the acute (4 days) and long-term (4 weeks) spheroid exposure datasets. For each gene, the optimal segmented regression model was selected using the lowest Bayesian Information Criterion (BIC). Model fitting parameters included a maximum of three breakpoints, a minimum of five samples per segment, and a breakpoint significance threshold of p < 0.2, consistent with the recommended Trendy settings. Genes identified by Trendy were interpreted as exhibiting distinct dose-responsive expression patterns, including monotonic, threshold-like, and non-monotonic responses.

### 2.4 Transcriptomic point-of-departure (tPOD) analysis

Transcriptomic point-of-departure (tPOD) analysis was performed using BMDExpress3.0 (Phillips et al., 2019; Yang et al., 2007) (BMDExpress - Sciome) to quantify concentration-dependent transcriptional potency of GenX exposure in HepG2/C3A spheroids. Acute (4 days) and long-term (4 weeks) exposure datasets were analyzed independently to determine whether exposure duration alters transcriptomic response and pathway-level potency. The CPM-normalized gene expression data were imported into BMDExpress3.0 for benchmark concentration modeling. Genes were prefiltered using Williams trend test and fold-change criteria to identify transcripts exhibiting concentration-dependent responses. Genes passing the prefiltering step were modeled using Bayesian Model Averaging with Hill, Power, Exponential 3, and Exponential five models. A benchmark response corresponding to one standard deviation from the control mean was applied. Modeled genes were subsequently filtered to remove poorly fitted or unreliable benchmark concentration estimates, including genes with benchmark concentrations greater than the highest tested concentration and genes with excessive uncertainty based on BMCU (Upper)/BMCL (Lower) ratios (O’Brien et al., 2025). Genes passing all pre- and post-filtering criteria were retained for downstream tPOD analysis. For each exposure duration, the tPOD was determined using the 25th-lowest-ranked gene BMD approach. Gene-level BMD estimates passing all pre- and post-filtering criteria were sorted in ascending order, and the BMD corresponding to the 25th-ranked gene was selected as the tPOD. This procedure was applied separately to the 4-day and 4-week exposure datasets. The resulting tPODs were expressed as nominal GenX concentrations in μM and compared descriptively to assess differences associated with exposure duration (Reardon et al., 2023).

## 3 Results

### 3.1 GenX-induced transcriptional responses are both dose- and time-dependent, with unexpected reversals in DEG magnitude across doses

Figure 1A summarizes the overall experimental approach used in this study. We used a ClinoStar bioreactor system (CelVivo) to continuously rotate HepG2/C3A spheroids generated by pre-aggregation procedure (see Methods), supporting prolonged cultures. The HepG2/C3A spheroids were then exposed to GenX, either acute (4 days) or long-term (4 weeks) from environmentally relevant (0.08 µM) to mechanistically informative concentrations (50 µM). To place this range in a human exposure context, the lowest nominal concentration, 0.08 µM, corresponds to approximately 26.4 ng/mL (GenX). This concentration falls within the overall serum concentration range reported in a Canadian study, although it exceeds the 95th percentile of 16.76 ng/mL; two participants had concentrations of 57 and 62 ng/mL (Schlezinger et al., 2025). In Wilmington, North Carolina, a community affected by industrial contamination of drinking water, serum GenX concentrations reached 15 ng/mL (Max), approximately 1.8-fold below our lowest test concentration (Cheng et al., 2025). These comparisons provide context for the lower end of our experimental range, rather than demonstrating that the entire range represents environmentally-relevant exposure. The higher (gradually increasing) concentrations support characterization of responses (i.e., mechanistically informative) across a broader sublethal experimental range (Addicks et al., 2025; Rowan-Carroll et al., 2025).

**FIGURE 1:**
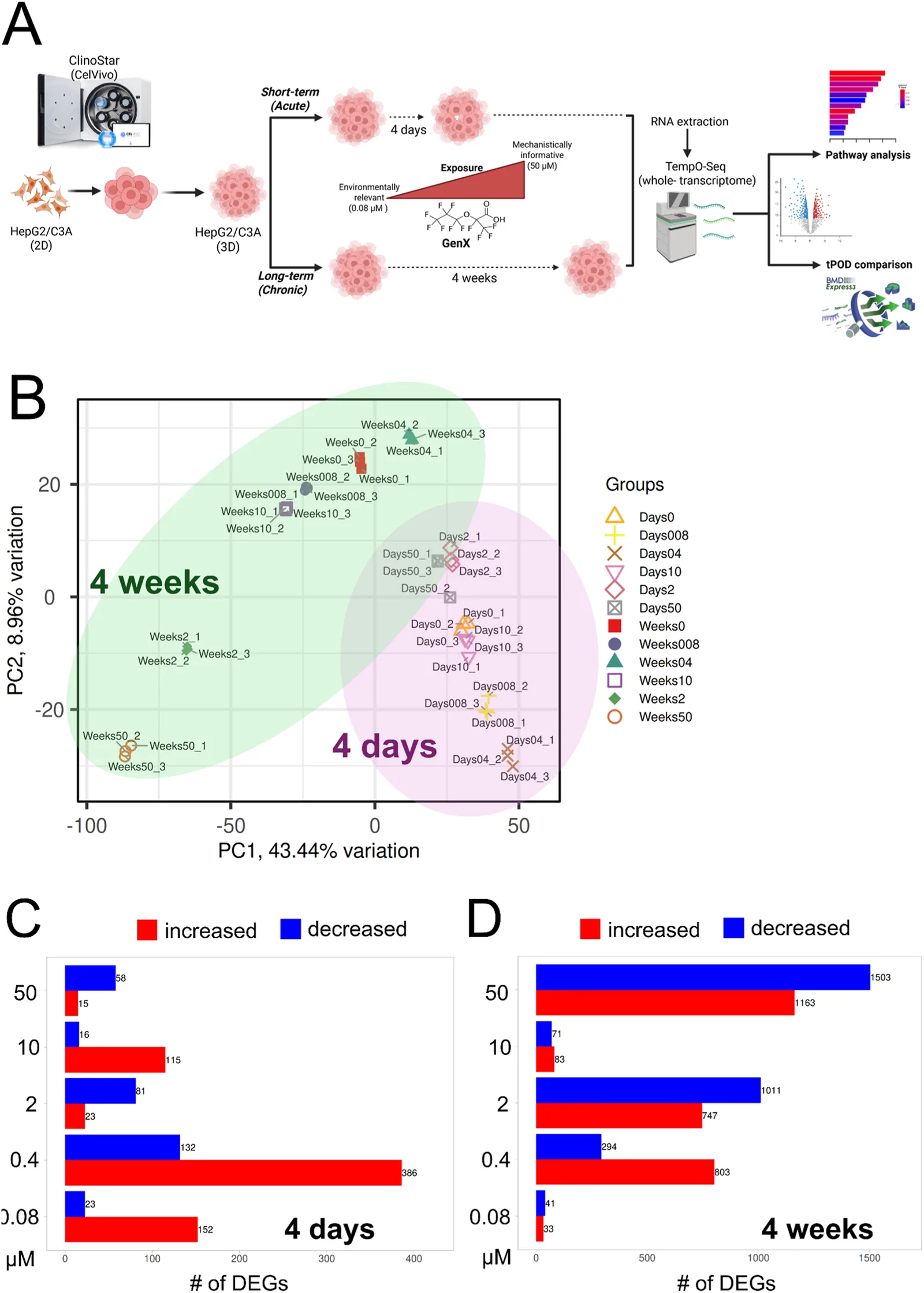
Experimental design and global transcriptional overview. **(A)** Schematic experimental design of this study. 3D spheroids generated using HepG2/C3A cells were exposed to GenX for 4 days (acute) or 4 weeks (4 weeks) at different doses from an environmentally relevant dose (0.08 μM) to a mechanistically informative dose (50 μM). Collected samples were used for TempO-Seq (transcriptome profiling), followed by pathways-level enrichment and tPOD comparison analyses. **(B)** PCA plot of all samples analyzed in this study. **(C,D)** Comparison of differentially expressed genes (DEGs) in acute (4 days) and long-term (4 weeks) GenX exposed HepG2/C3A spheroids across doses (DEGs criteria: Log_2_FC > 1 and FDR<0.05).

After exposures, RNAs from both dose-dependent acute and long-term samples were used for whole-transcriptome profiling *via* TempO-Seq followed by pathway enrichment and tPOD comparison analyses (Figure 1A). ATP-based cell viability was assessed in 3D HepG2/C3A spheroids using CellTiter-Glo after 96 h of GenX exposure at concentrations ranging from 0 to 400 μM (Supplementary Figure S1). Mean assay signals at 50, 75, and 100 μM were 144.1%, 145.7%, and 143.8% of the 0 μM control, respectively. Thus, the signal was not reduced at 50 μM, the highest concentration used for transcriptomic profiling. At 150 and 200 μM, mean signals were 96.0% and 97.5% of control, respectively, whereas higher concentrations of 300 and 400 μM reduced the signals to 64.8% and 42.6% of control.

Dimension reduction of the data from all of spheroid RNA samples of both acute and long-term GenX exposure *via* PCA revealed that acute and long-term sets are clearly separated (Figure 1B). Notably, long-term exposure samples are more separated from each other than the acute counterparts (Figure 1B). HepG2/C3A 3D spheroids exposed to GenX for 4 days or 4 weeks exhibited varying numbers of differentially expressed genes (DEGs) across GenX concentrations (0.08 μM–50 μM). For acute exposure to GenX, DEGs of 0.08 μM, 0.4 μM, 2 μM, 10 μM, 50 μM were 161 (Up: 142, Down: 19), 476 (Up: 349, Down: 127) 103 (Up: 15, Down: 88), 87 (Up: 71, Down: 16), and 65 (Up: 6, Down: 59), respectively (Figure 1C). Following acute exposure, the number of DEGs peaked at 0.4 μM and decreased at higher concentrations (Figure 1C), which is defined as a dose-response curve in which the slope changes sign at least once over the range of doses examined (More et al., 2021; van der Most et al., 2024). Following the long-term exposure, DEG counts increased from 0.08 to 2 μM, declined at 10 μM, and reached their maximum at 50 μM (Figure 1D). DEGs of 0.08 μM, 0.4 μM, 2 μM, 10 μM, 50 μM were 59 (Up: 32, Down: 27), 983 (Up: 789, Down: 294), 1711 (Up: 749, Down: 962), 157 (Up: 97, Down: 60), and 2656 (Up: 1168, Down: 1488), respectively (Figure 1D). Together, these results demonstrate that exposure duration influences both the extent of differential gene expression and the distribution of DEG counts across GenX concentrations.

### 3.2 Conserved GenX-responsive genes exhibit distinct transcriptional dynamics during acute and long-term exposure

To identify changes in mRNA level consistently associated with GenX exposure, regardless of concentration, we focused on genes that were differentially expressed (either increased or decreased mRNA levels) across all exposure concentrations within each exposure duration. These constitutively responsive genes were further analyzed using a modified application of Trendy analysis (Bacher et al., 2018) to characterize their expression dynamics across the concentration gradient (see Methods). We identified eight constitutively increased GenX-responsive transcripts and 9 decreased transcripts in acute exposure (Figure 2), while 132 increased and 186 decreased transcripts in long-term exposure (Figure 3).

**FIGURE 2:**
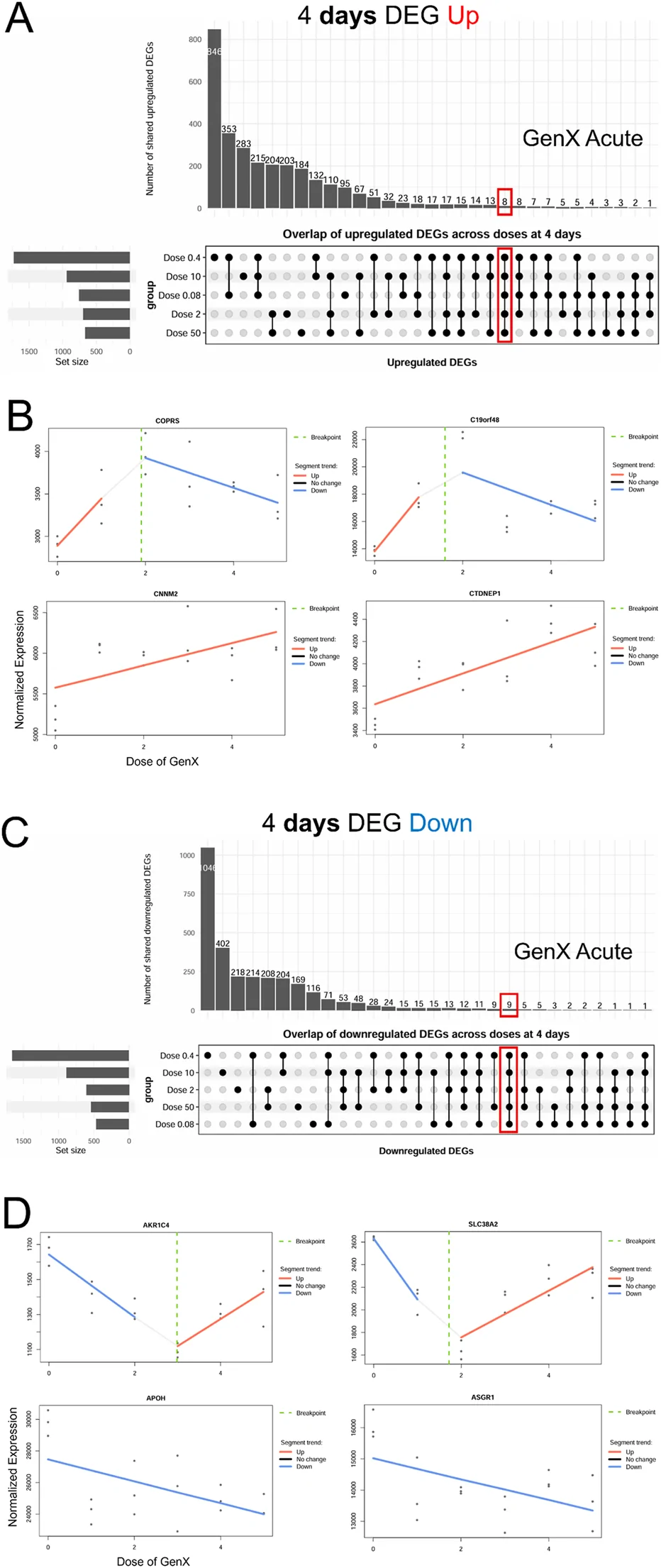
Conserved mRNA changes across GenX concentrations during acute (4 days) exposure condition. UpSet plots **(A,C)** showing the overlap of significantly altered genes (either increased (“Upregulated”) or decreased mRNA expression (“Downregulated”)) among HepG2/C3A spheroids exposed to GenX for 4 days at 0.08, 0.4, 2, 10, and 50 μM. Horizontal bars indicate the total number of differentially expressed genes (DEGs) (FDR <0.05) identified at each concentration, whereas vertical bars represent the number of genes shared among the indicated dose combinations. Constitutively altered transcripts across all GenX concentrations during acute exposure were subjected to Trendy segmented regression analysis (Bacher et al., 2018). Representative expression trajectories of selected transcripts are shown across the GenX concentration range **(B,D)**. Expression values are presented as normalized counts. Solid lines indicate fitted segmented regression models generated using Trendy.

**FIGURE 3:**
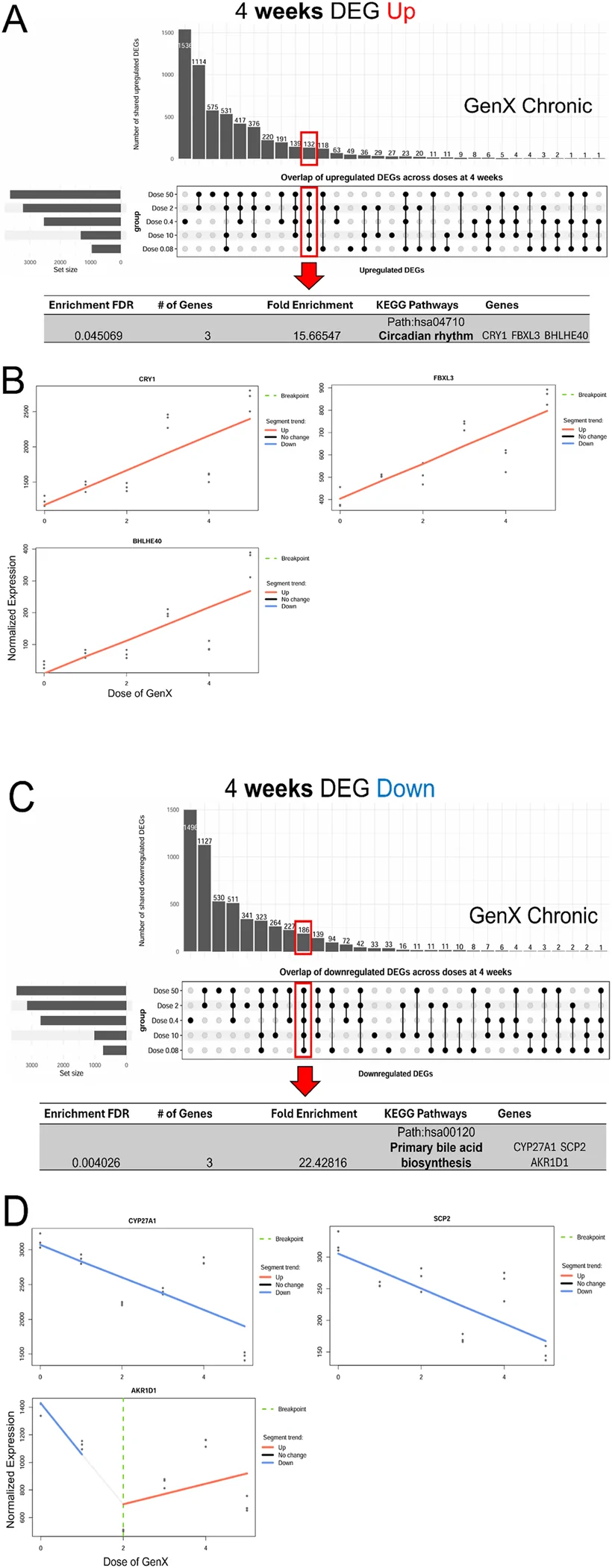
Conserved mRNA changes responses across GenX concentrations during long-term (4 weeks) exposure condition. UpSet plots **(A,C)** showing the overlap of significantly altered transcripts (either increased (UP DEGs) or decreased (DOWN DEGs) mRNA expression) among HepG2/C3A spheroids exposed to GenX for 4 weeks at 0.08, 0.4, 2, 10, and 50 μM. Horizontal bars indicate the total number of differentially expressed genes (DEGs) (FDR <0.05) identified at each concentration, whereas vertical bars represent the number of transcripts shared among the indicated dose combinations. Constitutively altered transcripts across all GenX concentrations during long-term exposure were subjected to Trendy segmented regression analysis (Bacher et al., 2018). Representative expression trajectories of selected transcripts are shown across the GenX concentration range **(B,D)**. Expression values are presented as normalized counts. Solid lines indicate fitted segmented regression models generated using Trendy.

Exploratory Trendy analysis of selected transcripts identified different expression trajectories across the concentration range (Figures 2B,D). *COPRS* and *C19orf48* showed an initial increase followed by a decline, whereas *CNNM2* and *CTDNEP1* showed sustained increases (Figure 2B). Among the decreased transcripts, *AKR1C4* and *SLC38A2* showed an initial decrease followed by partial recovery at higher concentrations, while *APOH* and *ASGR1* showed sustained decreases (Figure 2D). These examples illustrate gene-specific concentration–response patterns but do not quantify the prevalence of non-monotonic responses across the transcriptome.

The selected transcripts examined following long-term exposure exhibited predominantly increasing or decreasing expression trajectories across the tested concentrations (Figure 3). Of note, because the long-term exposure has substantially more DEGs, we first sought enriched biological pathways among those DEGs. For the increasing trajectory DEGs, circadian rhythm was the top KEGG enriched pathway, allowing us to prioritize the member genes (*CRY1*, *FBXL3*, and *BHLHE40*) (Figures 3A,B). For decreasing trajectory DEGs, primary bile acid biosynthesis was identified as a top KEGG enriched pathway, and we focused on member genes (*CYP27A1*, *SCP2*, and *AKR1D1*) for the Trendy analysis (Figures 3C,D). Trendy analysis of long-term increased transcripts revealed significant enrichment of the circadian rhythm pathway (Figure 3A). Notably, core circadian regulators, including *CRY1, FBXL3*, and *BHLHE40,* exhibited progressive increases across the GenX concentration range (Figure 3B), suggesting that long-term GenX exposure induces a coordinated transcriptional or other mRNA regulatory program involving circadian clock regulation. These findings further support the notion that exposure duration fundamentally influences the molecular response landscape and may determine whether early adaptive responses transition into sustained biological reprogramming. Consistently decreased transcripts across increasing GenX concentrations during the long-term exposure condition identified a coordinated set of transcripts encoding cellular components involved in primary bile acid biosynthesis that exhibited dose-dependent alterations in mRNA levels (Figure 3C). Notably, *CYP27A1* and *SCP2* displayed progressive decreases in expression, whereas *AKR1D1* exhibited a biphasic response characterized by an initial decrease followed by partial recovery at higher concentrations (Figure 3D). Given the central roles of the corresponding gene products in cholesterol turnover, intracellular sterol transport, and bile acid biosynthesis (Russell, 2003), these patterns collectively suggest that disruption of bile acid homeostasis represents a sensitive molecular response to long-term GenX exposure. Collectively, although concentration-specific mRNA changes dominated both exposure durations, long-term exposure produced substantially larger sets of transcripts shared across multiple concentrations, suggesting convergence toward stable mRNA changes during prolonged GenX exposure.

### 3.3 Acute low-dose GenX exposure drives robust changes in gene expression in HepG2/C3A spheroids

We next characterized concentration-dependent biological responses following acute GenX exposure using KEGG and Gene Ontology Biological Process (GO-BP) enrichment analyses of gene products from differentially expressed genes (Figure 4). We inferred distinct biological changes across the concentration range, revealing a progressive transition from mRNA changes consistent with a cell proliferative response at low concentrations to more metabolic and signaling perturbations at higher concentrations. At environmentally relevant concentrations (0.08 and 0.4 µM), enriched pathways were dominated by cell cycle-related processes. KEGG analysis identified cell cycle-associated pathways among genes whose mRNA expression levels increased, while GO-BP analysis showed significant enrichment for chromosome segregation, mitotic nuclear division, and cell cycle progression, indicating an early adaptive transcriptional response or other mRNA regulatory mechanisms (Figures 4A,B). Additional GO-BP enrichment analysis using STRING-DB at 0.08 µM further supported these findings, highlighting interconnected biological processes involved in mitotic cell cycle regulation, chromosome organization, and cell division, confirming that low-dose GenX primarily perturbs proliferative regulatory networks.

**FIGURE 4:**
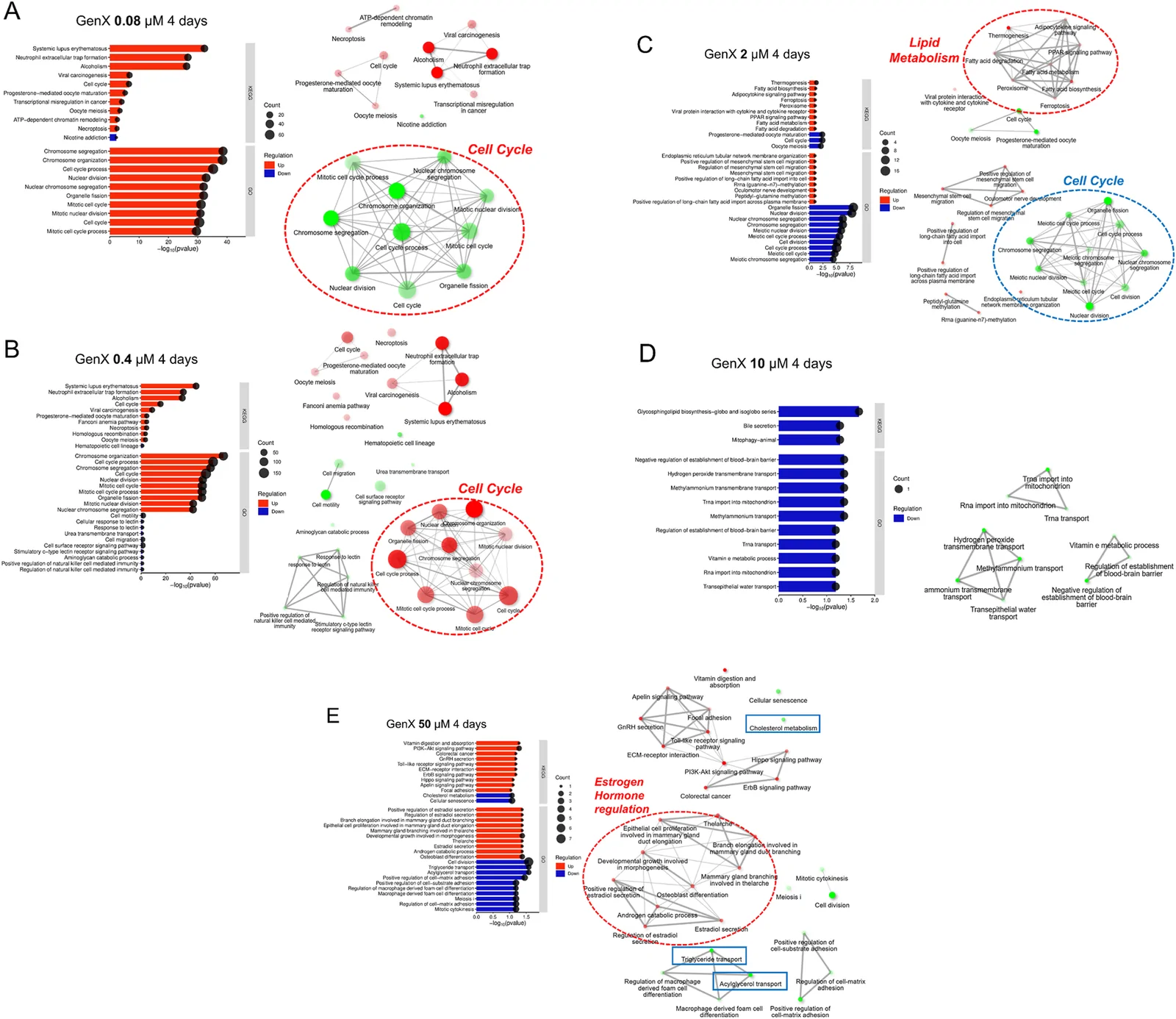
Acute GenX exposure induces concentration-dependent mRNA changes consistent with a transition from cell cycle regulation to lipid metabolism and endocrine-related pathways. Functional enrichment analysis of differentially expressed genes following 4 days of GenX exposure at 0.08 **(A)**, 0.4 **(B)**, 2 **(C)**, 10 **(D)**, and 50 **(E)** μM, respectively. Representative enriched KEGG and Gene Ontology Biological Process (GO-BP) terms are shown for each concentration with graphical presentation of clustered KEGG pathways and GO-BP. For the lowest concentration (0.08 μM), additional GO-BP enrichment analysis generated using STRING-DB is also presented. Enriched pathways were identified using DEGs (|log2FC| > 1, FDR < 0.05). Only the top 10 enriched pathways are shown.

At the intermediate concentration (2 µM), pathway enrichment of the protein products of the DEGs shifted toward lipid metabolism while maintaining enrichment of cell cycle-related processes, suggesting the onset of metabolic perturbation accompanying continued cell-cycle responses (Figure 4C). In contrast, relatively few significantly enriched pathways were detected at 10 µM despite measurable mRNA changes, consistent with a transient reduction in coordinated pathway-level responses (Figure 4D). At the highest concentration (50 µM), pathway enrichment shifted toward endocrine- and development-associated biological processes, including regulation of hormone levels and hormone metabolic processes, together with increased expression of components of PI3K-Akt, Toll-like receptor, and Hippo signaling pathways and suppression of cholesterol metabolism pathways (Figure 4E). Collectively, these findings demonstrate that acute GenX exposure induces concentration-dependent biological responses, characterized by a progressive transition from early mRNA changes encoding proteins involved in cell cycle regulation at low concentrations to lipid metabolic perturbation and, ultimately, stress- and endocrine-associated signaling pathways at higher concentrations.

### 3.4 Long-term (4 weeks) GenX exposure reveals *qualitatively* distinct transcriptional programs

Functional enrichment analysis of proteins encoded by DEGs following 4 weeks of GenX exposure demonstrated a progressive concentration-dependent remodeling of biological processes that differed markedly from the acute response (Figure 5). In contrast to acute exposure, where low concentrations primarily affected cell cycle-associated pathways, long-term exposure initiated coordinated mRNA changes consistent with metabolic alterations even at the lowest concentration tested, indicating increased biological response to prolonged GenX exposure.

**FIGURE 5:**
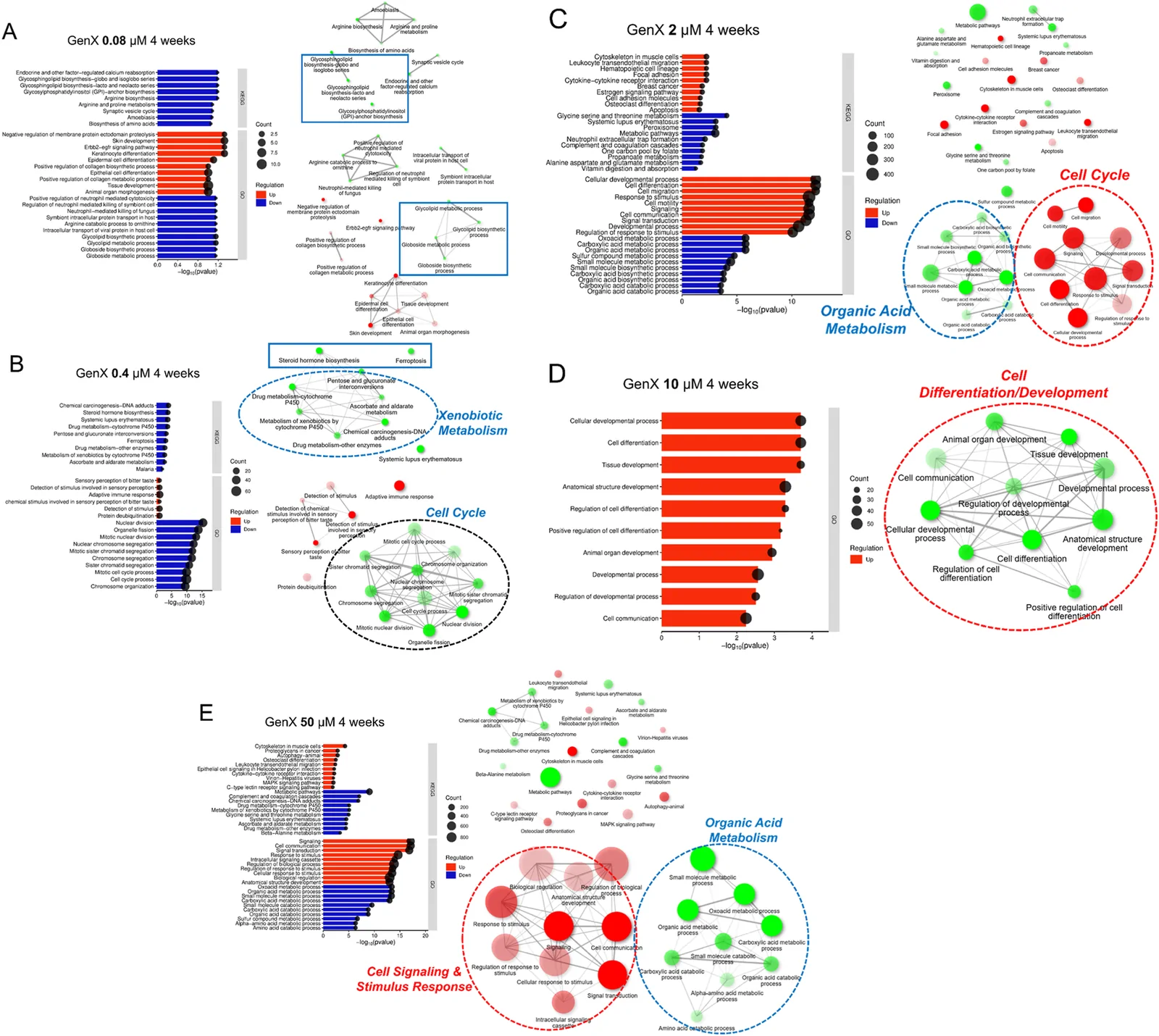
Long-term GenX exposure progressively shifts biological responses toward metabolic reprogramming and cellular signaling. Functional enrichment analysis of differentially expressed genes following 4 weeks of GenX exposure at 0.08 **(A)**, 0.4 **(B)**, 2 **(C)**, 10 **(D)**, and 50 **(E)** μM, respectively. Representative enriched KEGG and Gene Ontology Biological Process (GO-BP) terms are shown for each concentration with graphical presentation of clustered KEGG pathways and GO-BP. Enriched pathways were identified using DEGs (|log2FC| > 1, FDR < 0.05). Only the top 10 enriched pathways are shown.

At 0.08 µM, enrichment analysis revealed mRNA changes consistent with suppression of lipid metabolism-related pathways, including steroid hormone biosynthesis and glycosphingolipid biosynthesis, together with changes in amino acid metabolism, suggesting that prolonged exposure to environmentally relevant GenX concentrations was sufficient to perturb hepatic metabolic homeostasis (Figure 5A). At 0.4 µM, metabolic pathway remodeling became more pronounced, with additional enrichment of fatty acid metabolism and xenobiotic-related pathways, indicating expansion of the metabolic response beyond the lowest exposure concentration (Figure 5B). At the intermediate concentration (2 µM), broader perturbation of lipid metabolism and cellular metabolic processes was observed, accompanied by increasing enrichment of pathways involved in cellular signaling and homeostatic regulation (Figure 5C).

Although relatively fewer significantly enriched pathways were detected at 10 μM, similar to the acute response, extensive transcriptional alterations remained evident, suggesting transient attenuation of coordinated pathway enrichment despite substantial gene expression changes (Figure 5D). At the highest concentration (50 µM), long-term GenX exposure induced widespread suppression of transcripts encoding components of multiple metabolic pathways, including cytochrome P450-mediated xenobiotic metabolism, amino acid metabolism, and lipid metabolism, along with enrichment of multiple signaling pathways associated with cellular stress and structural remodeling (Figure 5E). Overall, long-term GenX exposure produced a coordinated concentration-dependent transcriptional reprogramming characterized by an early disruption of metabolic homeostasis at environmentally relevant concentrations followed by broad metabolic suppression and increased expression of cellular signaling pathways at higher concentrations.

### 3.5 Exposure duration drives progressive transcriptomic divergence following GenX exposure

To evaluate whether acute transcriptional responses could predict long-term outcomes, we performed correlation analyses comparing acute (4 days) and long-term (4 weeks) transcriptomic profiles across matched exposure concentrations (Supplementary Figure S2). We first evaluated correlations among biological replicates within each exposure condition and demonstrated excellent reproducibility, with Pearson correlation coefficients exceeding 0.99 for both control (0 μM) and high-dose (50 μM) groups (Supplementary Figures S2A,B; Supplementary Figures S2D,E). To assess the intrinsic stability of the steady state mRNA expression levels of the monocellular HepG2/C3A spheroid model over time, untreated control samples collected after 4 days and 4 weeks were compared. Control spheroids maintained a highly stable transcriptional profile throughout the culture period, exhibiting a strong correlation (R = 0.98) between time points (Supplementary Figure S2C). These findings indicate that prolonged culture alone had minimal impact on global gene expression patterns. In contrast, GenX exposure introduced a progressive temporal divergence that became increasingly apparent at higher concentrations. At 50 μM, the correlation between acute and long-term transcriptomic profiles decreased relative to untreated controls (R = 0.95), accompanied by increased dispersion of individual genes from the diagonal distribution (Supplementary Figures S2D,F). Although the overall correlation remained high, the increased scatter indicates that prolonged exposure resulted in substantial mRNA changes beyond the initial acute response.

These findings demonstrate that long-term GenX exposure does not simply amplify acute mRNA changes but instead induces a distinct mRNA expression profile that emerges over time. Together with the differential pathway enrichment and DEG analyses, the correlation results identify exposure duration as an independent determinant of GenX-induced transcriptomic changes and support the importance of incorporating long-term exposure models into GenX hazard assessment.

### 3.6 Benchmark dose modeling reveals time-dependent expansion of transcriptional responsiveness

To evaluate the influence of exposure duration on GenX-induced transcriptional responses, benchmark dose (BMD) distributions were compared between acute (4 days) and long-term (4 weeks) exposures using cumulative accumulation plots generated by BMDExpress 3.0 (Figure 6). Long-term exposure produced a marked upward shift in the accumulation curves compared with the acute condition, indicating that substantially more genes met benchmark dose modeling criteria. Differences were also evident in the distribution of BMD estimates. The long-term exposure curves were shifted toward lower BMD, benchmark dose lower confidence limit (BMDL) (Supplementary Figure S3A) and benchmark dose upper confidence limit (BMDU) (Supplementary Figure S3B) values than the acute exposure, indicating that a larger proportion of transcriptional responses occurred at lower dose ranges. In addition, the long-term condition exhibited a steeper accumulation pattern across intermediate BMD values, reflecting a greater concentration of responsive genes within a relatively narrow benchmark dose range.

**FIGURE 6:**
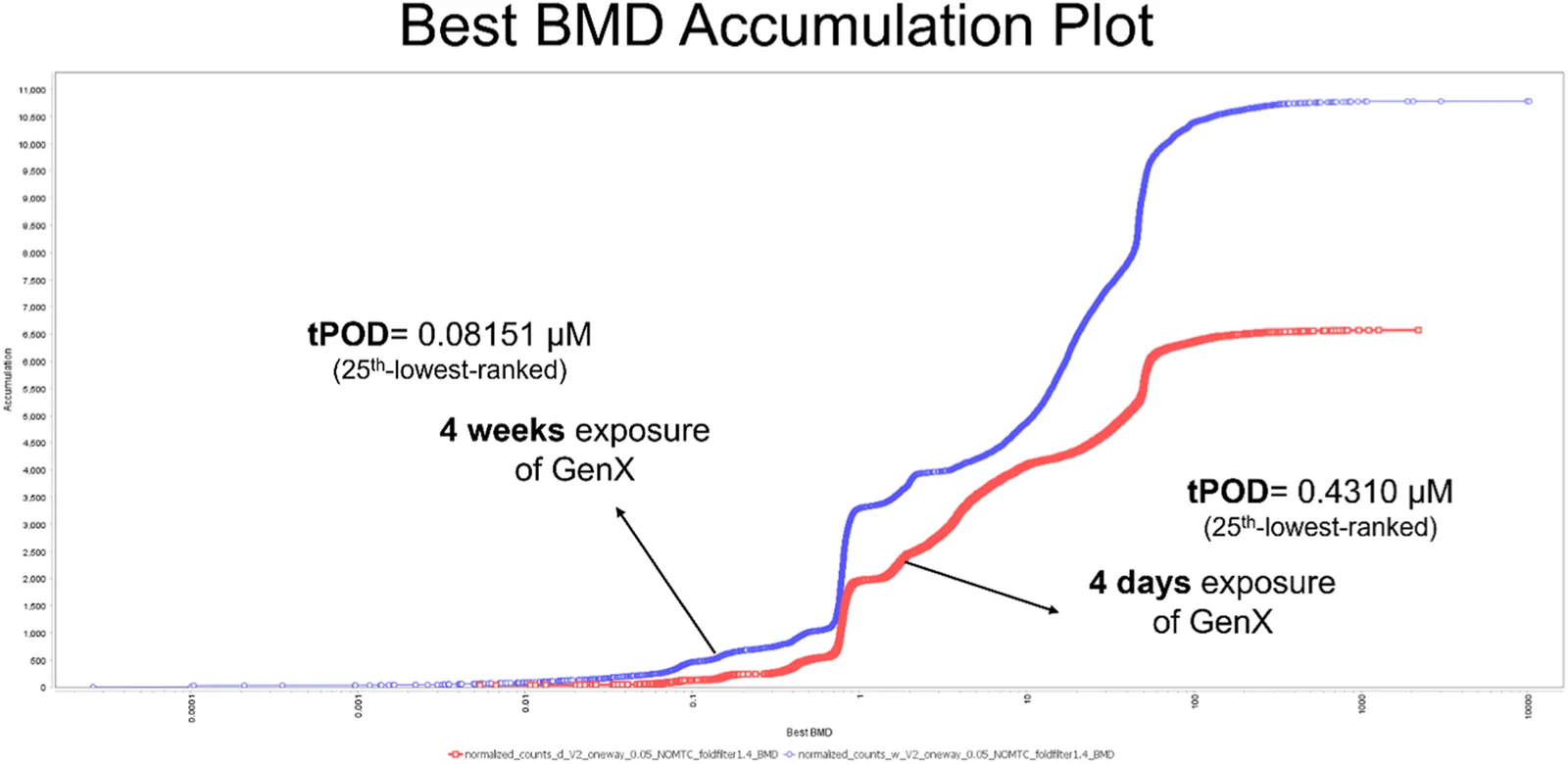
Gene-level benchmark dose (BMD) accumulation analyses for acute and long-term GenX exposure. BMD generated from transcriptomic benchmark dose modeling of GenX-exposed 3D HepG2/C3A spheroids is shown in the accumulation plot. Curves represent the cumulative number of genes meeting benchmark dose modeling criteria across increasing BMD values. Red lines indicate acute exposure (4 days), and blue lines indicate long-term exposure (4 weeks). The tPOD, defined as the 25th-lowest-ranked gene BMD, was 0.4310 µM after 4 days and 0.08151 µM after 4 weeks. Benchmark dose modeling was performed using normalized gene expression data and the best-fitting model selected for each gene according to BMDExpress criteria (O’Brien et al., 2025).

In contrast, acute exposure produced a more gradual accumulation profile and a smaller overall number of modeled genes (i.e., the number of accumulated genes presented in y-axis of Figure 6). The higher plateau observed following long-term exposure further demonstrates a broader transcriptional response after prolonged GenX treatment. Using the 25th-lowest-ranked gene BMD approach, the tPOD was 0.4310 μM after 4 days and 0.08151 μM after 4 weeks. Thus, the 4-week tPOD was approximately 5.29-fold lower than the 4-day estimate. These results demonstrate an exposure-duration-associated difference in the rank-based tPOD within the HepG2/C3A spheroid model.

Collectively, these findings indicate that exposure duration substantially influences the distribution and extent of GenX-induced transcriptional responses, with long-term exposure resulting in a larger number of benchmark dose-responsive genes and a distinct BMD distribution compared with acute exposure.

## 4 Discussion

The present study provides systematic comparisons of acute and long-term GenX exposure in a monocellular 3D HepG2/C3A hepatic spheroid model across environmentally relevant and mechanistically informative concentrations. The principal findings were that exposure duration shaped the magnitude and biological character of GenX-induced transcriptomic responses, with long-term exposure eliciting broader metabolic perturbations and lower transcriptomic BMD estimates; and the long-term exposure elicited broader metabolic perturbations and lower transcriptomic BMD estimates. The long-term responses therefore differed in both magnitude and biological character from those observed after short-term exposure. Together, these findings highlight the importance of considering both concentration and exposure duration when interpreting transcriptomic indicators of GenX activity and their potential application in human health risk assessment.

One of the most notable findings was the pronounced non-concentration dependent response following acute exposure, with the largest number of differentially expressed genes at 0.4 μM rather than the highest tested concentration. Similar behavior has been reported for PFAS and other environmental contaminants (Alijagic et al., 2024; Chu et al., 2023; Dunder et al., 2023; Lagarde et al., 2015; Park et al., 2021). This pattern indicates that the breadth and nature of low- and intermediate-concentration transcriptomic responses cannot necessarily be predicted from high-concentration data alone. Acute responses also differed in their biological composition: low concentrations primarily affected cell-cycle-associated gene expression, whereas intermediate concentrations preferentially altered lipid metabolism and PPAR signaling. STRING-DB network analysis supported the involvement of interconnected processes related to mitotic regulation, chromosome organization, and cell division. At the highest concentration, enriched pathways included PI3K-Akt, ErbB, and Toll-like receptor signaling. PI3K-Akt signaling regulates cellular survival and proliferation and has been implicated in PFAS-related responses (Glaviano et al., 2023; Wan et al., 2026). Collectively, these findings suggest engagement of different regulatory programs across the concentration range. However, pathway enrichment alone does not establish whether these changes represent adaptation, altered proliferation, or functional impairment.

In contrast, long-term exposure elicited broader metabolic perturbations, with lipid-related pathways affected even at the lowest concentration. Changes in glycosphingolipid, steroid, fatty acid, and peroxisomal pathways are consistent with evidence implicating disrupted lipid homeostasis in PFAS toxicity (Fenton et al., 2021; Shi et al., 2024). At higher concentrations, suppression of diverse metabolic pathways coincided with enrichment of inflammatory and stress-response signaling. These findings suggest altered hepatic metabolic regulation, although a loss of hepatocellular identity cannot be established from pathway enrichment alone. Similar metabolic and fibroinflammatory transcriptional signatures have been reported in GenX-exposed primary human hepatocytes (Robarts et al., 2022), providing a point of comparison with the present model. The long-term exposure also increased the number of transcripts consistently altered across all tested concentrations. This finding is consistent with a shared molecular response to sustained GenX treatment. Among these transcripts, increased expression of circadian rhythm genes is noteworthy because clock regulators are closely linked to hepatic metabolism, xenobiotic responses, and lipid homeostasis (Bolshette et al., 2023; DeBruyne et al., 2014). Altered clock-associated gene expression may therefore contribute to the broader metabolic remodeling observed after prolonged exposure. Together, these results support the emergence of sustained transcriptional programs that differ from the acute response.

The enrichment of cell-cycle-related pathways following both 4-day and 4-week GenX exposure indicates that cell-cycle-associated gene expression was affected at both sampled time points. However, shared pathway enrichment does not establish sustained activation or a uniform functional effect across concentrations and exposure durations. In the same 3D hepatic model, the 96-h (4 days) CellTiter-Glo assessment showed no reduction in ATP-based viability signal across the transcriptomic concentration range. Thus, the acute cell-cycle-associated transcriptional changes were observed without a concomitant decrease in this viability endpoint.

The elevated ATP signals at intermediate concentrations may reflect changes in viable cell number, ATP content per cell, or both, and therefore do not demonstrate increased proliferation. These short-term measurements also do not establish viability following 4-week exposure. Direct measurements of DNA synthesis, cell-cycle distribution, and changes in cell number at the corresponding concentrations and exposure durations will be needed to determine the functional consequences of the transcriptional findings. The absence of such pathway-specific functional validation remains a limitation of the present study.

Perhaps the most important finding from a risk assessment perspective was the limited concordance between acute and long-term responses. Many pathways enriched after 4 weeks of exposure were absent or only weakly represented during acute exposure, and transcriptomic correlation analyses demonstrated increasing divergence between acute and long-term transcriptional states with increasing GenX concentration. These results suggest that long-term toxicity cannot be reliably inferred from short-term studies and instead reflects secondary and adaptive molecular programs that emerge during sustained exposure. Therefore, these findings suggest that exposure duration should be considered not only when interpreting transcriptomic mechanisms but also when applying tPODs in chemical risk assessment, as short-term transcriptomic responses may not fully reflect the coordinated metabolic reprogramming associated with long-term exposure. Our results also support the growing recognition that long-term low-dose PFAS exposure may engage unique biological processes that cannot be predicted from acute or high-dose studies alone (Zhang et al., 2025). Consistent with this interpretation, benchmark dose modeling (BMD) demonstrated that long-term exposure lowered transcriptomic points of departure and expanded the number of affected biological pathways, indicating that prolonged exposure increased transcriptomic response to GenX. That is, Long-term exposure resulted in a leftward shift of transcriptomic BMD distributions, suggesting increased transcriptional response and expanded biological engagement following prolonged GenX exposure. The approximately 5.29-fold lower 25th-ranked tPOD after 4 weeks compared to 4 days indicates that exposure duration influenced the rank-based estimate of transcriptional responsiveness to GenX in this model. Previous *in vitro* hepatocyte studies provide context for these findings. Heintz et al. (2024) reported a BMC as low as 15.1 μM for PPARα target genes in pooled primary human hepatocytes after 24 h of HFPO-DA exposure. Although our tPODs of 0.4310 and 0.08151 μM were numerically lower, these estimates summarize different endpoints and should not be interpreted as directly equivalent measures of potency (Heintz et al., 2022). As transcriptomic points of departure are increasingly incorporated into next-generation risk assessment (NRGA) framework (Auerbach et al., 2024), our findings suggest that tPODs derived from long-term 3D hepatic *in vitro* models may provide more biologically informative and health-protective estimates of chemical potency than those generated by conventional acute exposure systems. More broadly, the data indicate that prolonged GenX exposure unmasks molecular responses associated with metabolic dysfunction and loss of hepatocellular identity that are not evident during short-term exposure, highlighting the importance of incorporating exposure duration into PFAS hazard characterization and human health risk assessment. The regulatory relevance of these findings requires consideration of exposure scale and dosimetry. The EPA chronic oral reference dose for GenX chemicals is 3 × 10^−6^ mg/kg/day (US EPA, 2021), which cannot be directly compared with nominal *in vitro* concentrations expressed in μM. For context, the lowest concentration tested here, 0.08 μM, corresponds to approximately 26.4 μg/L of HFPO-DA acid, substantially exceeding the US EPA (2022) non-regulatory lifetime drinking-water health advisory of 10 ng/L (“Lifetime Drinking Water Health Advisories for Four Perfluoroalkyl Substances,” 2022). This comparison illustrates differences in exposure scale and does not constitute a margin-of-exposure calculation, because culture-medium concentrations and drinking-water concentrations do not represent equivalent internal doses. Translation of the transcriptomic responses to human exposure would require consideration of medium binding, cellular uptake, and human toxicokinetics. To our knowledge, there is no currently accepted PBPK model for GenX in people that could be utilized for *in vitro* to *in vivo* extrapolation so it is difficult to directly compare the *in vitro* exposures in this study with the expected *in vivo* exposures in people. Accordingly, this study provides evidence relevant to mechanistic hazard characterization rather than a quantitative estimate of risk at environmentally encountered exposure levels.

Several limitations of the model should be considered when interpreting these findings. The HepG2/C3A spheroids used here consist of a single hepatocyte-like cell type and therefore do not reproduce the full cellular composition or tissue architecture of the human liver. In particular, the absence of Kupffer cells and hepatic stellate cells precludes assessment of multicellular processes such as inflammatory amplification, immune-hepatocyte interactions, and fibrogenic responses (Messner et al., 2013; Prestigiacomo et al., 2017). Accordingly, enrichment of inflammatory or fibrosis-related pathways should be interpreted as transcriptional perturbation within HepG2/C3A cells rather than direct evidence of multicellular liver responses. Future studies should validate the key concentration- and duration-dependent findings in multicellular human liver spheroids or organoid systems containing hepatocytes, Kupffer cells, and stellate cells. The transformed phenotype of HepG2/C3A cells also limits extrapolation to normal adult hepatocytes. Compared with primary human hepatocytes, HepG2 cells differ in proliferative behavior and the expression and activity of hepatic drug-metabolizing enzymes (Wilkening et al., 2003). Although spheroid culture supports prolonged exposure studies, it does not eliminate these intrinsic differences. Accordingly, the cell-cycle-associated transcriptional changes observed here may reflect modulation of proliferative programs characteristic of HepG2/C3A cells, rather than equivalent responses in largely quiescent adult hepatocytes. Time-matched vehicle controls account for baseline culture conditions but do not establish that treatment responses are independent of cellular maturation or proliferative state. Primary human hepatocyte spheroids provide a relevant system for evaluating whether these responses extend to more differentiated hepatic cells (Bell et al., 2016).

## Data Availability

The datasets generated in this study are publicly available in the NCBI Gene Expression Om-nibus (GEO) under accession number GSE339046.
