# Supplementary material for: Exposure duration shapes GenX-induced transcriptomics responses in 3D HepG2/C3A spheroids
Source: Front Toxicol. 2026 Oct 14;8:1966541. doi: 10.3389/ftox.2026.1966541 (PMC13713026; doi:10.3389/ftox.2026.1966541)
Supplement: Supplementary file 1 [file DataSheet1.pdf]

iDEP: Pre-Processing and Exploratory Data Analysis

Code

- Show All Code
- Hide All Code

iDEP: Pre-Processing and Exploratory Data Analysis

If this site has contributed to your work, please cite our article:

Ge, S.X., Son, E.W. & Yao, R. iDEP: an integrated web application for differential expression and pathway analysis of RNA-Seq data. BMC Bioinformatics 19, 534 (2018). https://doi.org/10.1186/s12859-018-2486-6

Summary

Normalized expression values were analyzed using iDEP v2.5.0. The data was first filtered to remove genes below 0.5 in at least 1 sample. The data was not log transformed. Missing values were imputed using geneMedian.

After mapping, there are 19683 unique genes in 36 samples. Of the 19488 genes passed filter, 18992 were converted to Ensembl/STRING gene IDs in our database. The remaining 496 genes were kept in the data using original IDs.

```
if (params$data_file_format == 1) {
  total_counts_ggplot(
    counts_data = processed_data$raw_counts,
    sample_info = params$sample_info,
    type = "RAW",
    plots_color_select = params$plots_color_select
  )
} else {
  cat("Total counts plot requires raw count data (integer read counts). ",
      "Your data was loaded as a normalized/FPKM/TPM expression matrix, which
  does not have ",
      "meaningful total-count sums per sample.\n")
}
```

Total counts plot requires raw count data (integer read counts). Your data was loaded as a normalized/FPKM/TPM expression matrix, which does not have meaningful total-count sums per sample.

```
if (params$data_file_format == 1 && !is.null(processed_data$raw_counts)) {
  filtered <- as.data.frame(colSums(processed_data$raw_counts))
  original <- as.data.frame(colSums(params$loaded_data))
  df <- merge(x = original, y = filtered, by = "row.names")
  colnames(df) <- c("Sample", "Total Counts", "Total Counts After Filter")
  df$`Counts Removed` <- df$`Total Counts` - df$`Total Counts After Filter`
  numeric_cols <- setdiff(names(df), "Sample")
```

```r
  df[numeric_cols] <- lapply(df[numeric_cols], function(col) {
    format(col, big.mark = ",", scientific = FALSE, trim = TRUE)
  })
  knitr::kable(
    df,
    caption = "Total reads per sample before and after filtering."
  )
} else if (params$data_file_format != 1) {
  cat("Read counts table not shown: filtering statistics are only meaningful for
  raw count data. ",
      "Your data was loaded as a normalized/FPKM/TPM expression matrix.\n")
} else if (is.null(processed_data$raw_counts)) {
  cat("Read counts table not shown: raw counts were not available after
  pre-processing.\n")
}
```

Read counts table not shown: filtering statistics are only meaningful for raw count data. Your data was loaded as a normalized/FPKM/TPM expression matrix.

Distributions

```r
eda_scatter(
  processed_data = processed_data$data,
  plot_xaxis = params$scatter_x,
  plot_yaxis = params$scatter_y
)
```

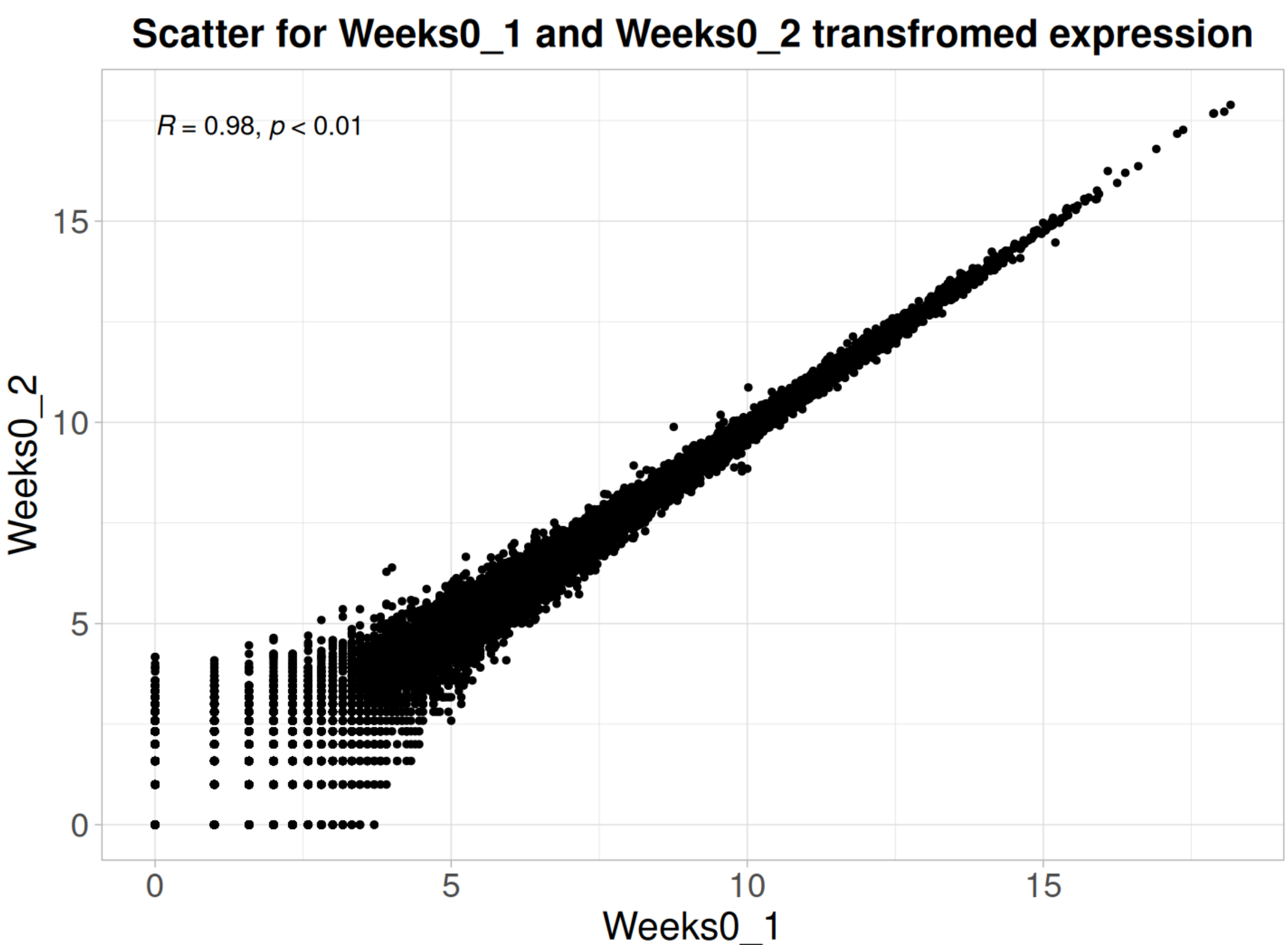

```r
eda_boxplot(
  processed_data = processed_data$data,
  sample_info = params$sample_info,
  plots_color_select = params$plots_color_select
)
```

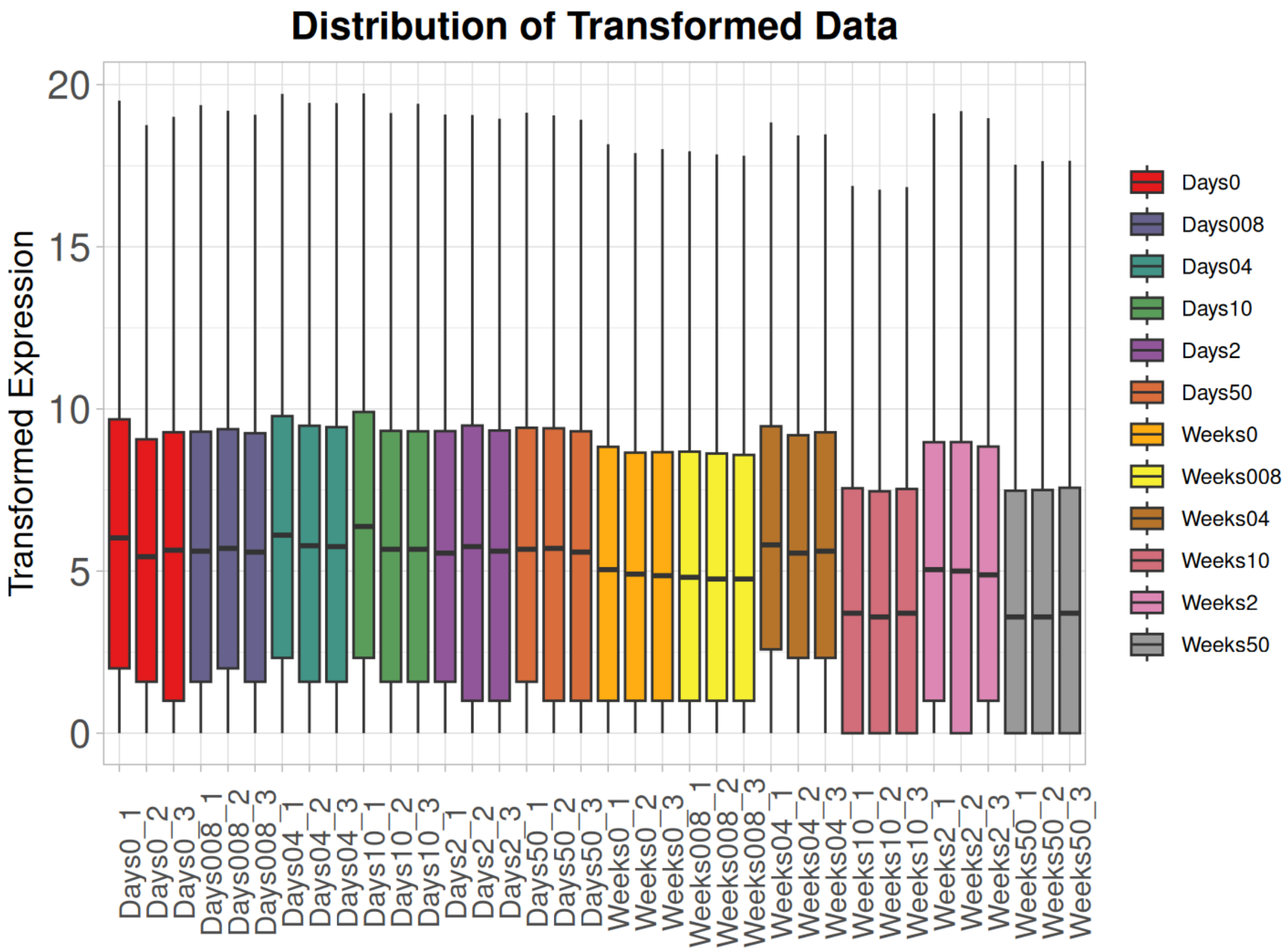


```r
eda_density(
  processed_data = processed_data$data,
  sample_info = params$sample_info,
  plots_color_select = params$plots_color_select
)
```

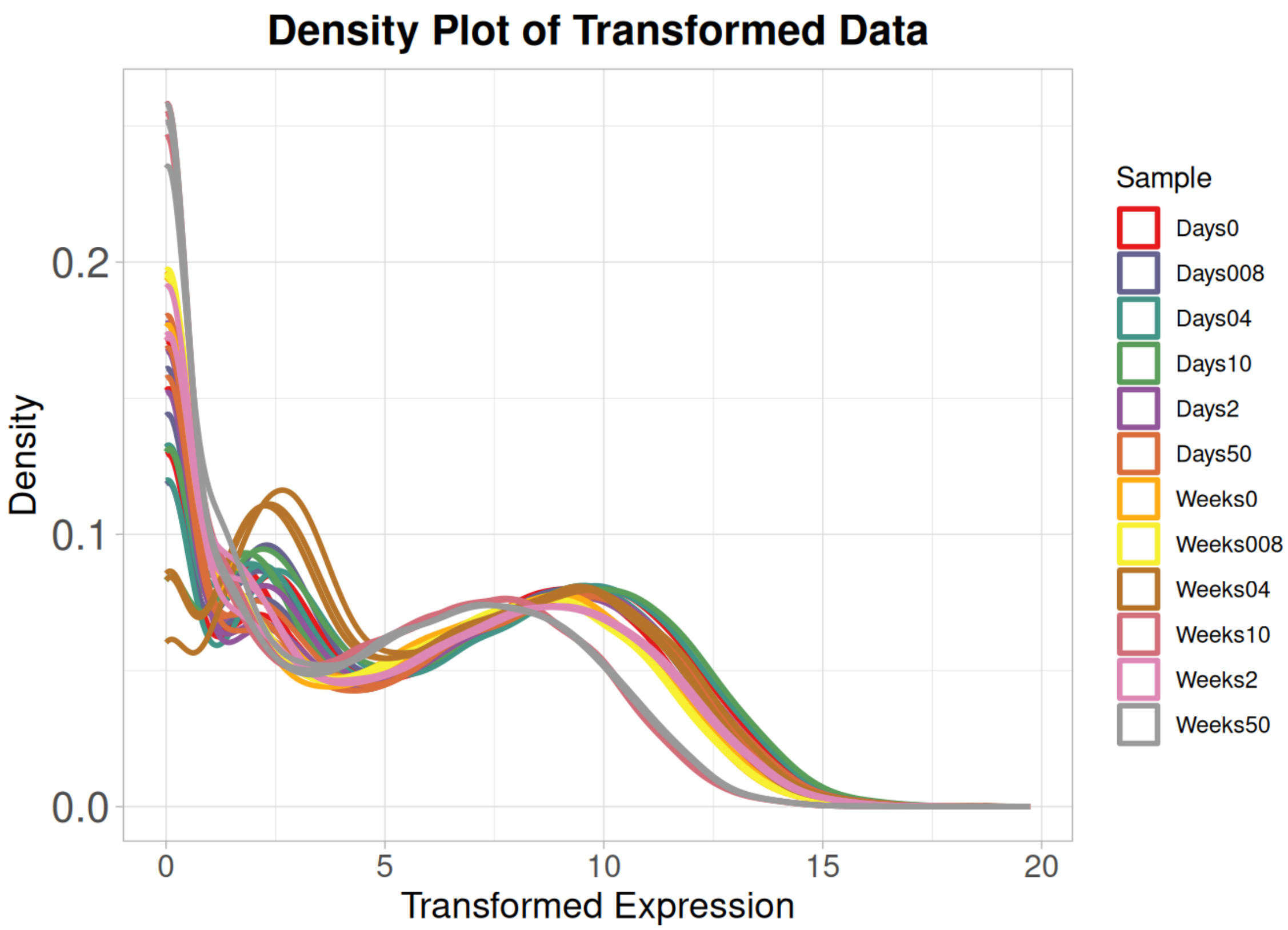


```
mean_sd_plot(
  processed_data = processed_data$data,
  heat_cols = params$sd_color,
  rank = params$rank
)
```

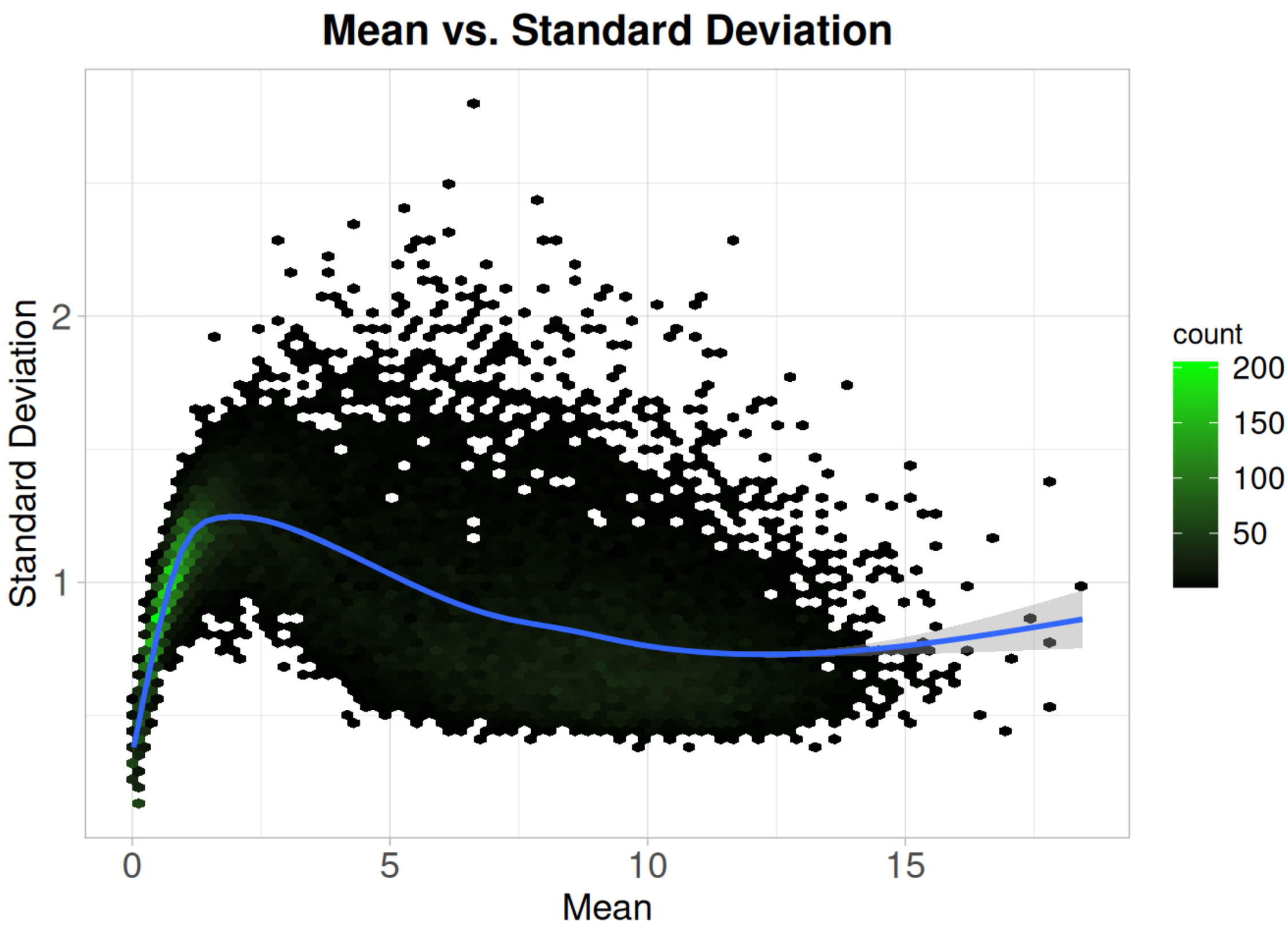


```
if (!is.null(params$all_gene_info) && "ensembl_gene_id" %in%
  colnames(params$all_gene_info)) {
  gene_counts_ggplot(
    counts_data = params$loaded_data,
    sample_info = params$sample_info,
    type = "Raw",
    all_gene_info = params$all_gene_info,
    plots_color_select = params$plots_color_select
  )
} else if (is.null(params$all_gene_info)) {
  cat("Gene biotype plot not shown: gene annotation data is unavailable. ",
      "This plot requires gene IDs that iDEP can map to Ensembl records.\n")
} else {
  cat("Gene biotype plot not shown: the gene annotation table does not contain an
  `ensembl_gene_id` column. ",
      "iDEP was unable to map your gene IDs to Ensembl records.\n")
}
```

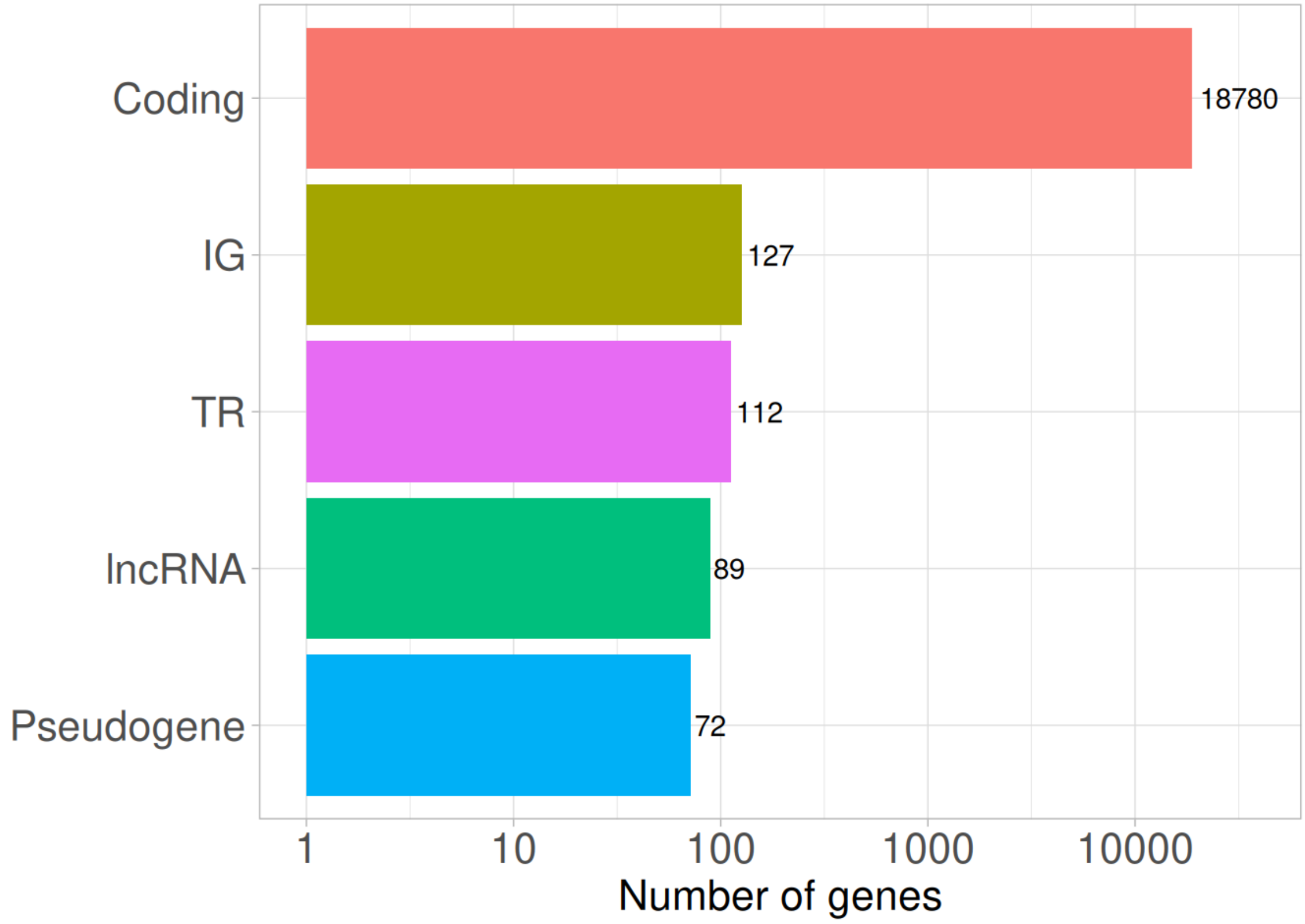

QC Plots

```
if (params$data_file_format == 1 && !is.null(params$all_gene_info) &&
  "ensembl_gene_id" %in% colnames(params$all_gene_info)) {
  rrna <- rRNA_counts_ggplot(
    counts_data = params$loaded_data,
    sample_info = params$sample_info,
    type = "Raw",
    all_gene_info = params$all_gene_info,
    plots_color_select = params$plots_color_select
  )
  print(rrna$plot)
  if (!is.null(rrna$warning)) {
    cat("\n\n**Warning:** ", rrna$warning, "\n")
  }
} else if (params$data_file_format != 1 && (is.null(params$all_gene_info) ||
  !"ensembl_gene_id" %in% colnames(params$all_gene_info))) {
  cat("rRNA/gene type QC plot requires both raw count data and ",
      "Ensembl-mappable gene IDs. Your data is normalized and gene IDs could not
  be mapped to Ensembl.\n")
} else if (params$data_file_format != 1) {
  cat("rRNA/gene type QC plot requires raw count data (integer read counts). ",
      "Proportions of rRNA and other biotypes are only interpretable from
  un-normalized counts.\n")
} else {
  cat("rRNA/gene type QC plot not shown: gene IDs could not be mapped to Ensembl
  records. ",
      "Provide Ensembl IDs or standard gene symbols.\n")
}
```

rRNA/gene type QC plot requires raw count data (integer read counts). Proportions of rRNA and other biotypes are only interpretable from un-normalized counts.

```
if (params$data_file_format == 1 && !is.null(params$all_gene_info) &&
  "ensembl_gene_id" %in% colnames(params$all_gene_info)) {
  use_boxplot <- isTRUE(params$chr_use_boxplot)
  chr_counts <- chr_counts_ggplot(
    counts_data = params$loaded_data,
    sample_info = params$sample_info,
    type = "Raw",
    all_gene_info = params$all_gene_info,
    plots_color_select = params$plots_color_select,
    use_boxplot = use_boxplot
  )
  print(chr_counts$plot)
  if (!is.null(chr_counts$warning)) {
    cat("\n\n**Warning:** ", chr_counts$warning, "\n")
  }
} else if (params$data_file_format != 1 && (is.null(params$all_gene_info) ||
  !"ensembl_gene_id" %in% colnames(params$all_gene_info))) {
  cat("Chromosome raw counts plot requires both raw count data and ",
      "Ensembl-mappable gene IDs for chromosomal assignment. Neither condition is
  met.\n")
} else if (params$data_file_format != 1) {
  cat("Chromosome raw counts plot requires raw count data. ",
      "Per-chromosome read distributions are not meaningful for normalized
  expression values.\n")
} else {
  cat("Chromosome raw counts plot not shown: gene IDs could not be mapped to
  Ensembl records.",
      "Provide Ensembl IDs or standard gene symbols.\n")
}
```

Chromosome raw counts plot requires raw count data. Per-chromosome read distributions are not meaningful for normalized expression values.

```
if (!is.null(processed_data$data) && !is.null(params$all_gene_info) &&
  "ensembl_gene_id" %in% colnames(params$all_gene_info)) {
  use_boxplot_norm <- isTRUE(params$chr_normalized_use_boxplot)
  chr_normalized <- chr_normalized_ggplot(
    counts_data = processed_data$data,
    sample_info = params$sample_info,
    type = "Transformed",
    all_gene_info = params$all_gene_info,
    plots_color_select = params$plots_color_select,
    use_boxplot = use_boxplot_norm
  )
  print(chr_normalized$plot)
  if (!is.null(chr_normalized$warning)) {
    cat("\n\n**Warning:** ", chr_normalized$warning, "\n")
  }
} else if (is.null(processed_data$data)) {
  cat("Chromosome normalized expression plot not shown: processed expression data
  is unavailable.\n")
} else {
```

```
  cat("Chromosome normalized expression plot not shown: gene IDs could not be
  mapped to Ensembl records",
      "Provide Ensembl IDs or standard gene symbols.\n")
}
```

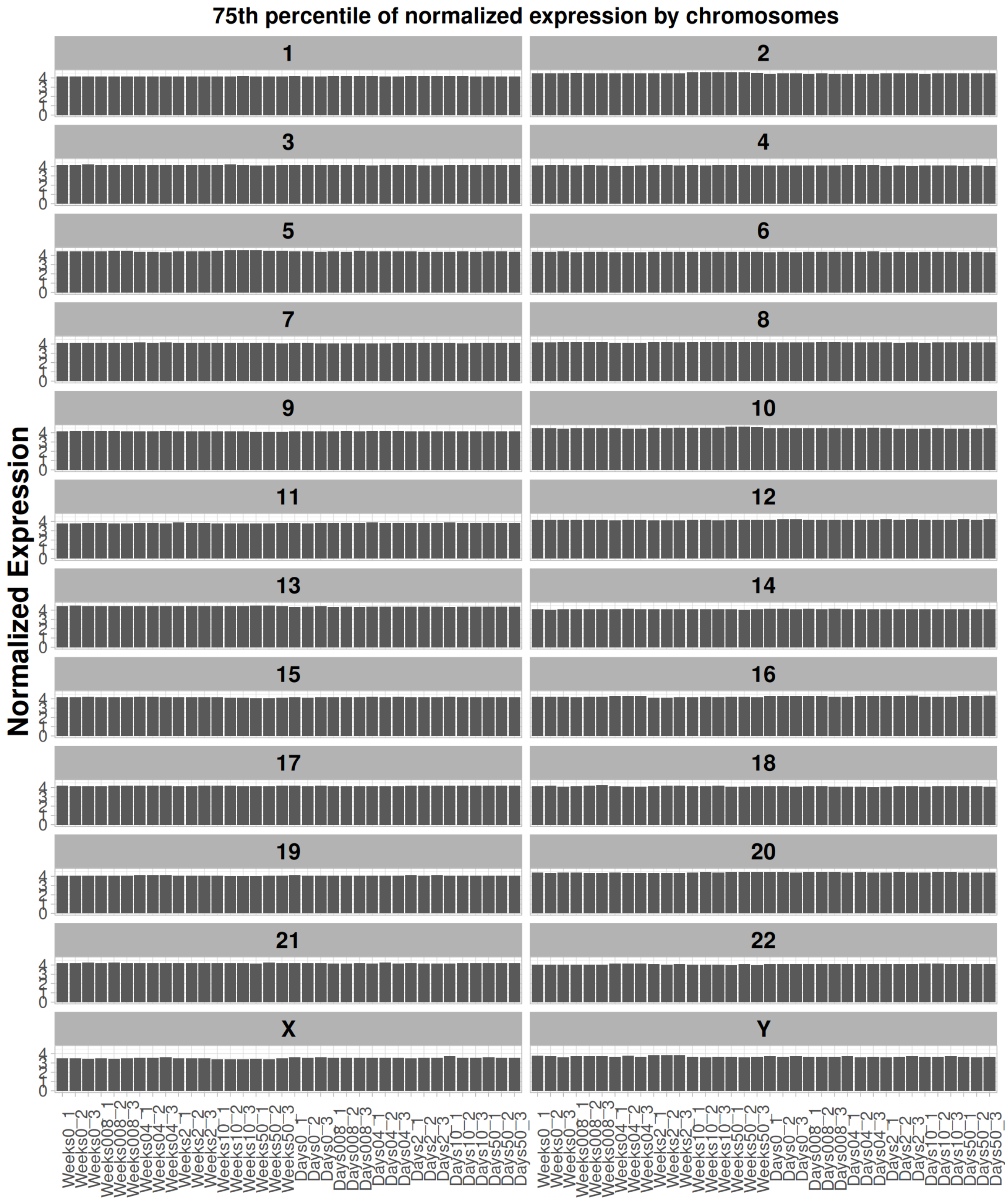

Marker Gene Plots

```
marker_payloads <- marker_gene_plot_payloads(
  expr_matrix = processed_data$data,
  sample_info = params$sample_info,
  raw_counts = processed_data$raw_counts,
  converted_data = params$loaded_data,
```

```r
  all_gene_info = params$all_gene_info
)

if (length(marker_payloads) == 0) {
  if (is.null(params$all_gene_info) || !"ensembl_gene_id" %in%
  colnames(params$all_gene_info)) {
    cat("Marker gene plots not shown: gene IDs could not be mapped to Ensembl
  records. ",
        "Provide Ensembl IDs or standard gene symbols.\n")
  } else {
    cat("Marker gene plots not shown: no marker genes from the reference sets
  were detected in this dataset.\n")
  }
} else {
  counts_flag <- !is.null(processed_data$raw_counts)
  for (payload in marker_payloads) {
    cat("#### ", payload$description, "\n\n", sep = "")
    plot_obj <- build_gene_expression_plot(
      expr_df = payload$data,
      display_name = payload$display_name,
      palette_name = params$plots_color_select,
      plot_type = "bar",
      data_type = "raw",
      counts_are_counts = counts_flag
    )
    plot_obj <- refine_ggplot2(
      p = plot_obj,
      gridline = params$plot_grid_lines,
      ggplot2_theme = params$ggplot2_theme
    )
    print(plot_obj)
    cat("\n\n")
  }
}
```

Housekeeping gene

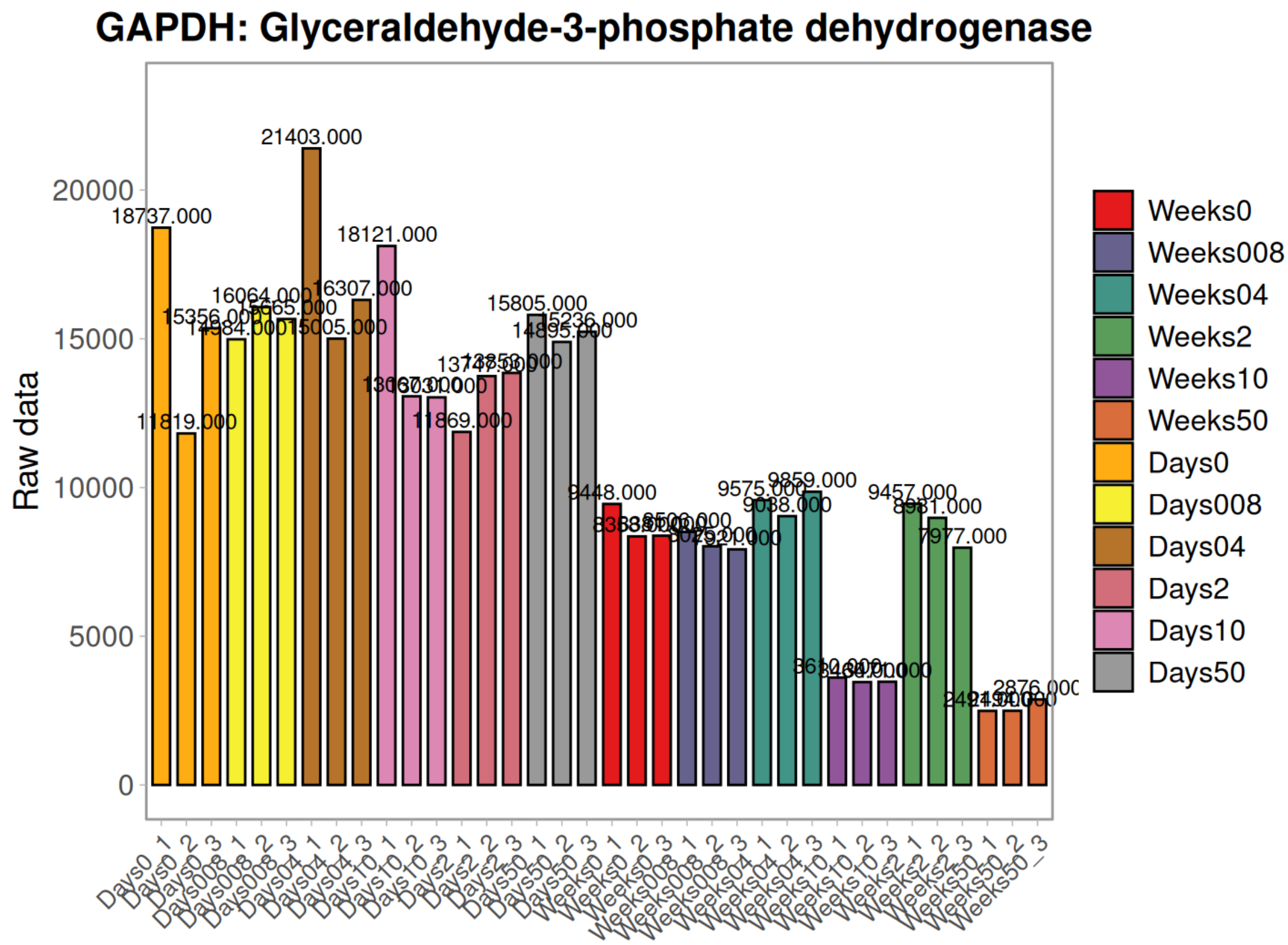

Housekeeping gene

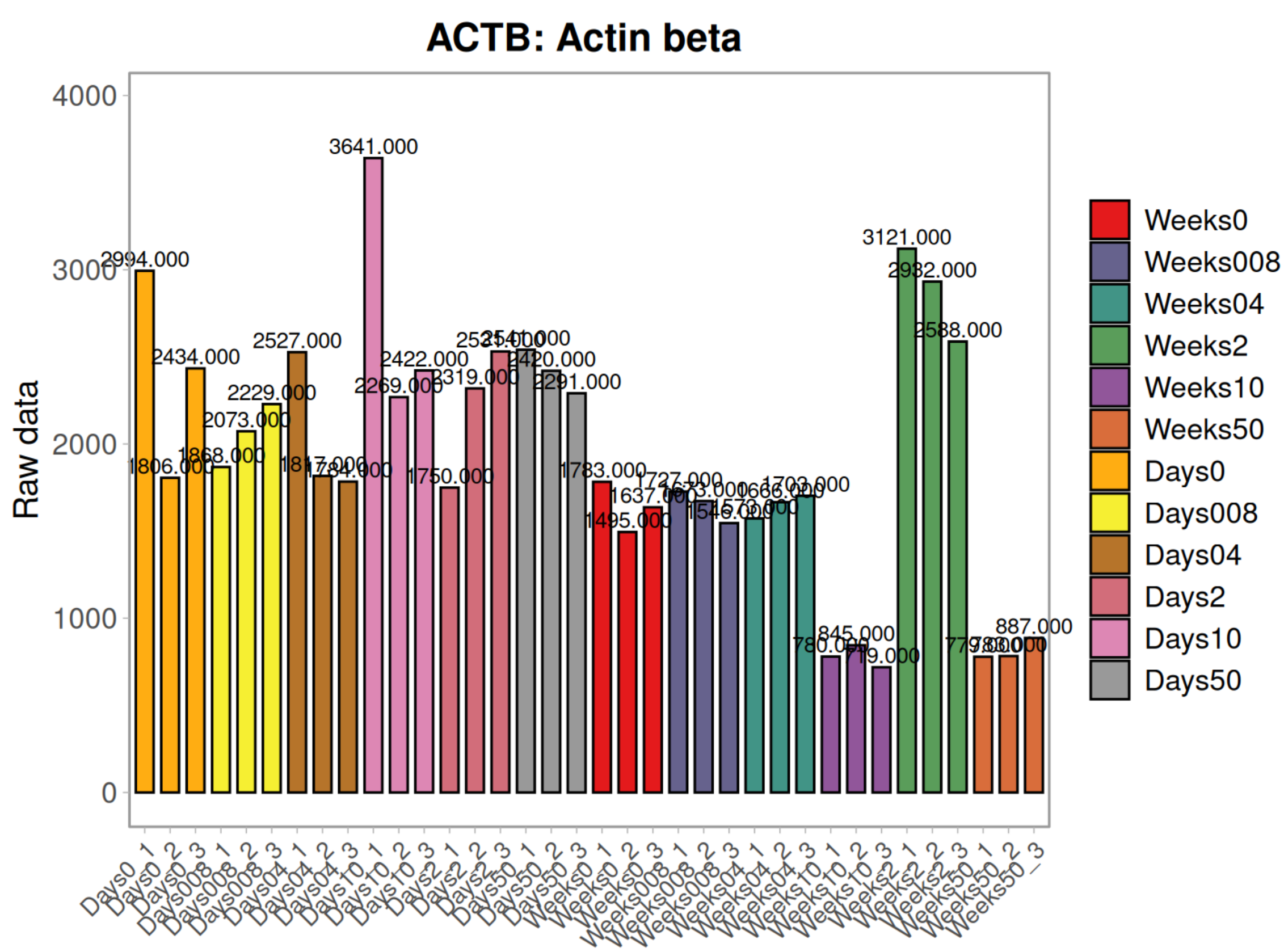

Histone mRNAs lack poly(A) tails

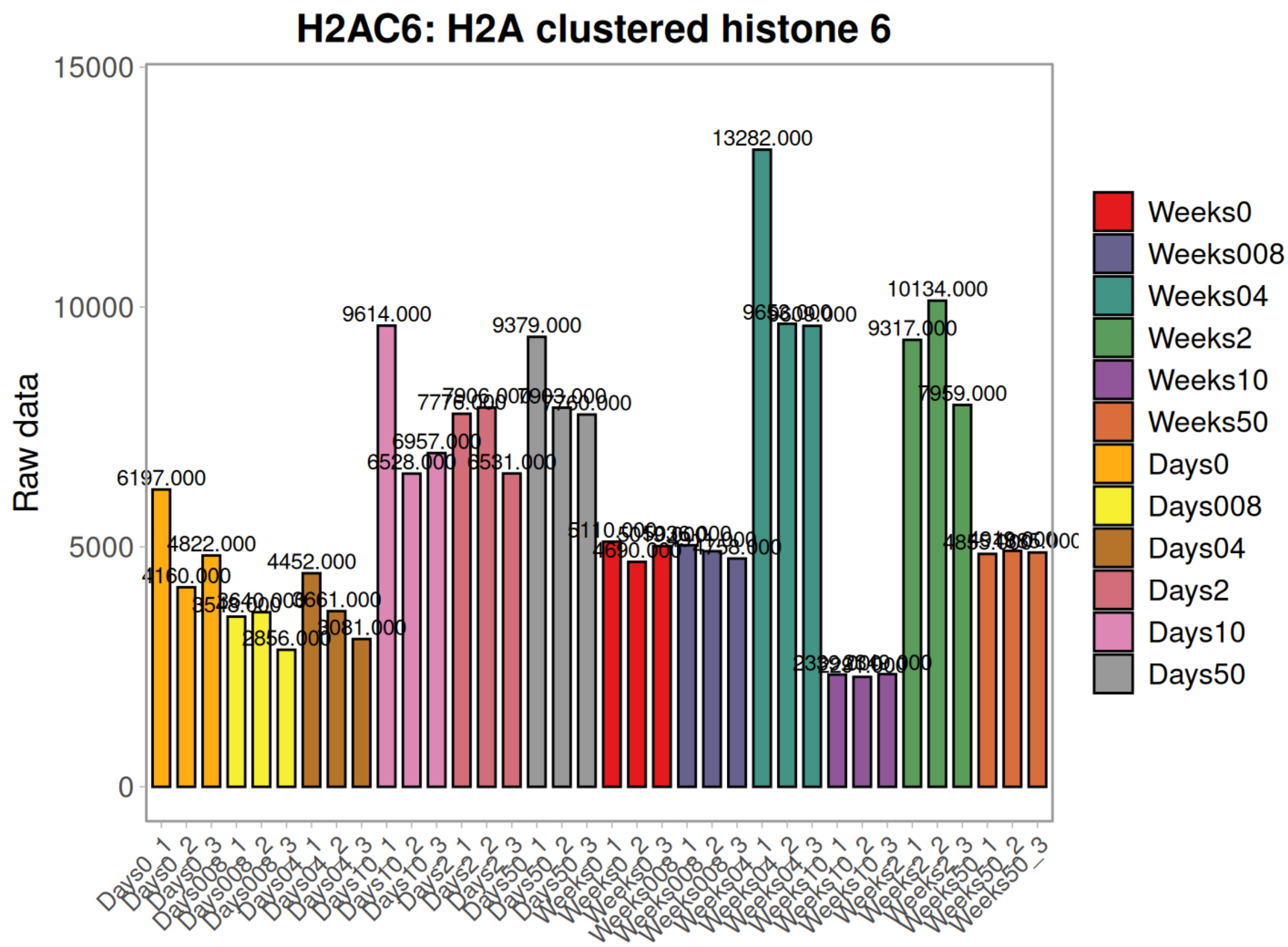

Male-specific

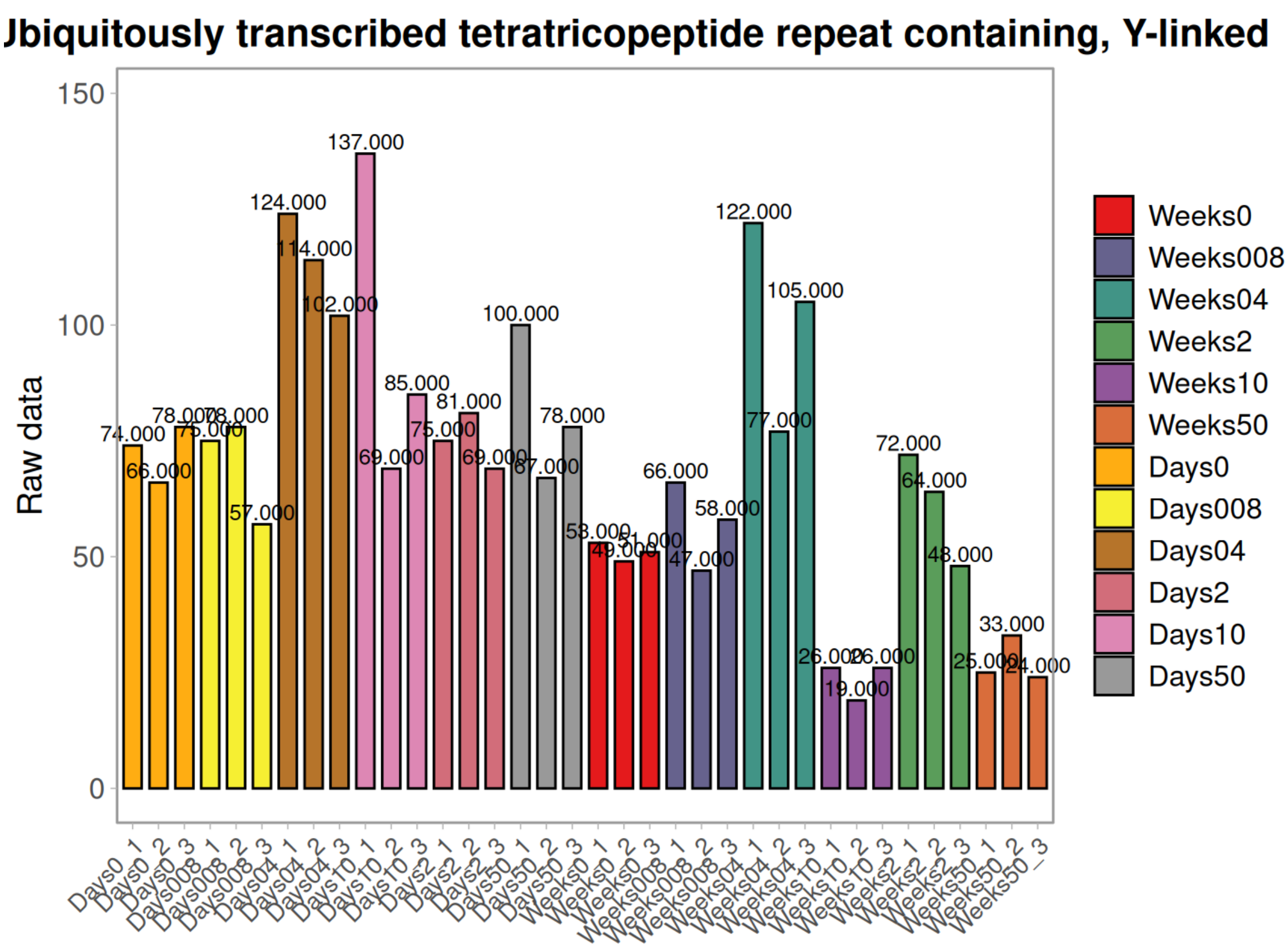

Female-specific

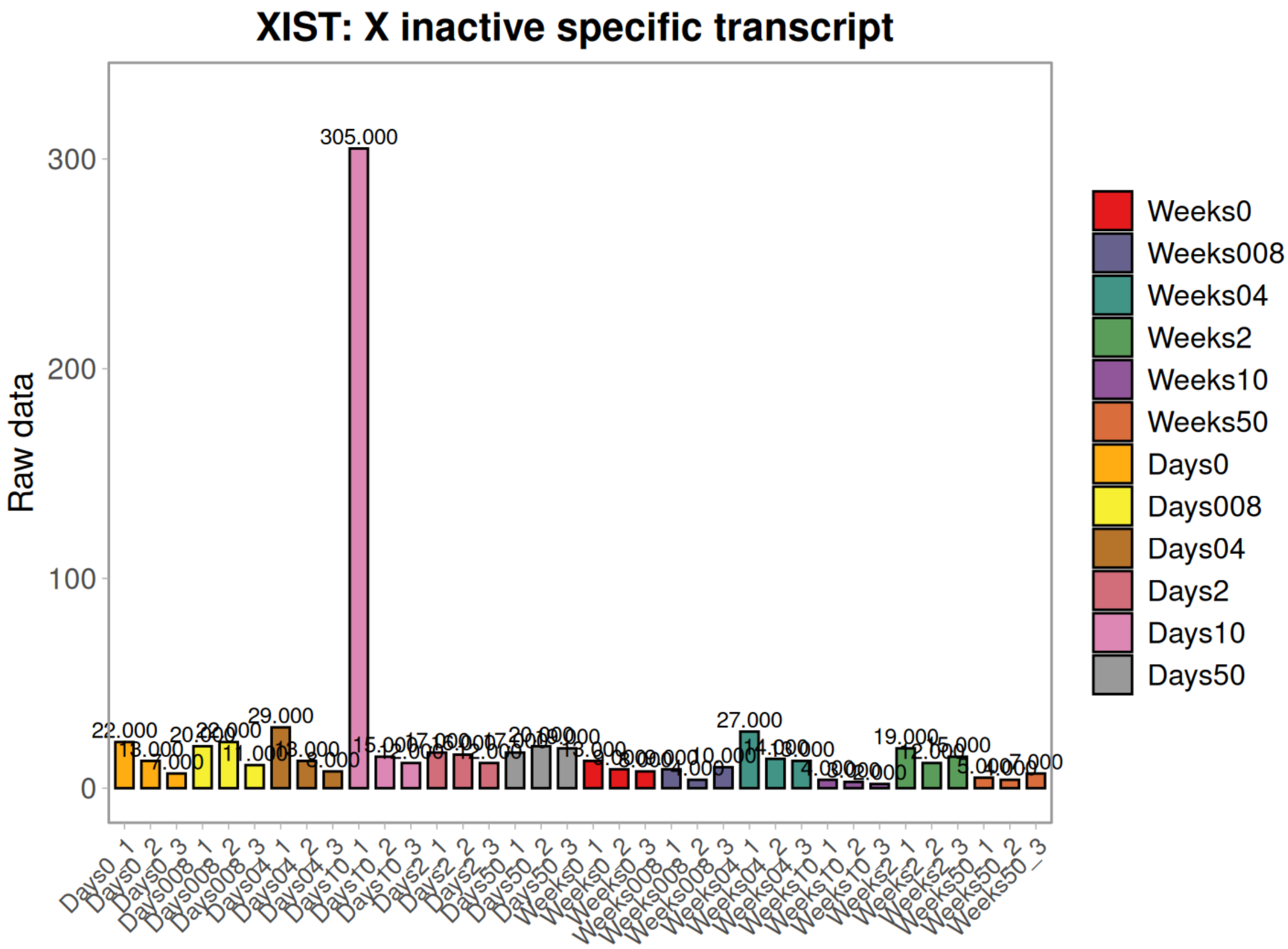


```
cat("\n<br>\n\n## Gene Plot\n\n")
```

Gene Plot

```
if (is.null(params$selected_gene) || length(params$selected_gene) == 0) {
  cat("No genes were available for plotting at render time.\n")
} else {
  p <- individual_plots(
    individual_data = params$individual_data,
    sample_info = params$sample_info,
    selected_gene = params$selected_gene,
    gene_plot_box = params$gene_plot_box,
    use_sd = params$use_sd,
    lab_rotate = params$lab_rotate,
    plots_color_select = params$plots_color_select,
    plot_raw = params$plot_raw,
    plot_tukey = params$plot_tukey
  )

  p <- refine_ggplot2(
    p = p,
    gridline = params$plot_grid_lines,
    ggplot2_theme = params$ggplot2_theme
  )

  print(p)
}
```

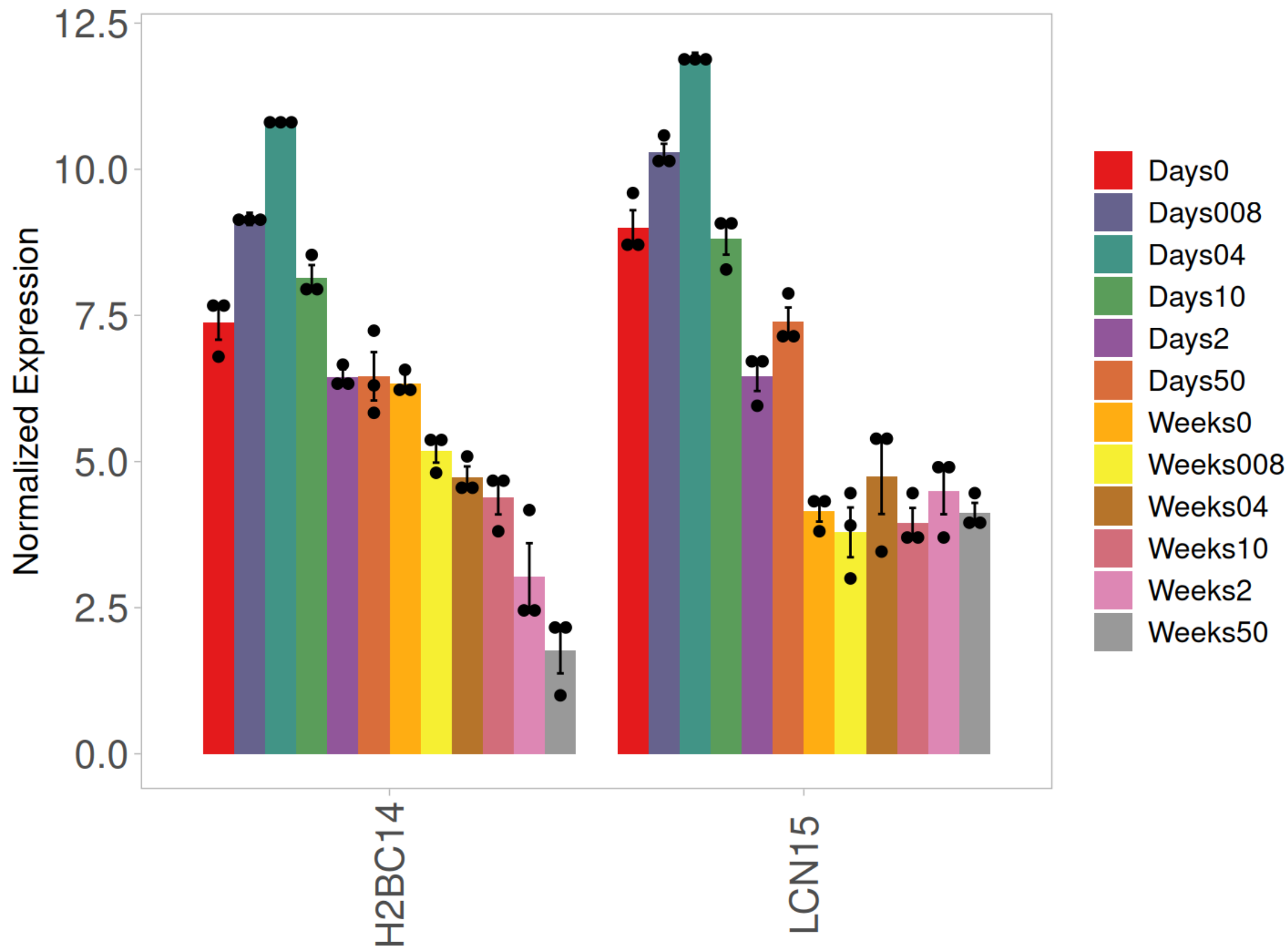

To Reproduce This Report Locally

1. Install the latest iDEP package (once per machine). All the functions and the Rmarkdown code are downloaded with the package. This pulls in ~355 dependencies and can take up to an hour the first time.

```
install.packages("pak")
pak::pkg_install("url::https://github.com/orditus-llc/idep-desktop/releases/
  download/v2.5.0/idep250_2.5.0.tar.gz")
```

Newer releases are listed at https://github.com/orditus-llc/idep-desktop/releases – use the `v*` tags, not the `desktop-v*` ones.

2. Download the RData file from the Prep tab.

3. Load the downloaded `.RData` workspace and render this report directly in R or RStudio:

```
load("idep_session_YYYY_MM_DD.Rdata")

# Read counts data saves report_params directly; the other input formats wrap
# everything in a single params_r list. This handles both.
if (exists("params_r")) report_params <- params_r$report_params

library(report_params$idep_package, character.only = TRUE)
rmarkdown::render(
  system.file("app/www/RMD", "pre_process_workflow.Rmd", package =
  report_params$idep_package),
  output_dir = getwd(),
  params = report_params,
  envir = new.env(parent = globalenv())
)
```

The resulting HTML will reproduce what you see in the Prep tab, using the saved `report_params` list bundled inside every `.RData` download.

Generated using iDEP v2.5.0 (https://bioinformatics.sdstate.edu/idep). 2026-09-01 21:56:25 UTC. Accuracy not guaranteed.
