# Supplementary material for: Exposure duration shapes GenX-induced transcriptomics responses in 3D HepG2/C3A spheroids
Source: Front Toxicol. 2026 Oct 14;8:1966541. doi: 10.3389/ftox.2026.1966541 (PMC13713026; doi:10.3389/ftox.2026.1966541)
Supplement: Supplementary file 2 [file Supplementaryfile1.docx]

Supplementary Material

**Exposure Duration Shapes GenX-Induced Transcriptomic Responses in 3D HepG2/C3A Spheroids**

**Chanhee Kim^1*^, Abderrahmane Tagmount^1^, Zhaohan Zhu^2^, Benjamin Aiken^1^, W. Brad Barbazuk^3,4^, Rhonda Bacher^2^, and Christopher D. Vulpe^1*^**

^1^Center for Human and Environmental Toxicology, Department of Physiological Sciences, College of Veterinary Medicine, University of Florida, Gainesville, FL, United States

^2^Department of Biostatistics, University of Florida, FL, United States

^3^Department of Biology, University of Florida, FL, United States

^4^University of Florida Genetics Institute, University of Florida, FL, United States

*** Correspondence:**
Chanhee Kim ([ch.kim@ufl.edu](mailto:ch.kim@ufl.edu)) & Christopher D. Vulpe (cvulpe@ufl.edu)

Supplementary Figures (Figure S1 and Figure S3)

**Figure S1**. Effect of GenX exposure on ATP-dependent viability signals in 3D HepG2/C3A spheroids. HepG2/C3A spheroids were exposed to GenX (0-400 µM) for 96 h (4-day). ATP-dependent luminescence was measured using the CellTiter-Glo® 3D Cell Viability Assay. Values were normalized to the vehicle-treated control (0 μM), which was set to 100%; the dashed line indicates the control level. Bars represent the mean ± SD, and open circles indicate individual replicates (n = 3 per concentration).

**Figure S2.** Correlation analysis of transcriptomic profiles across exposure durations and biological replicates. (A–C) Scatterplots showing pairwise correlations of transformed gene expression values under control (0 μM) conditions. (A) Correlation between biological replicates from the 4 days exposure group (4 days_0 μM_rep1 vs. 4 days_0 μM_rep2). (B) Correlation between biological replicates from the 4 weeks exposure group (4 weeks_0 μM_rep1 vs. 4 weeks_0 μM_rep2). (C) Correlation between representative samples from the 4 days and 4 weeks control groups (4 days_0 μM_rep1 vs. 4 weeks_0 μM_rep1). (D–F) Scatterplots showing pairwise correlations of transformed gene expression values under the highest GenX exposure condition (50 μM). (D) Correlation between biological replicates from the 4 days exposure group (4 days_50 μM_rep1 vs. 4 days_50 μM_rep2). (E) Correlation between biological replicates from the 4 weeks exposure group (4 weeks_50 μM_rep1 vs. 4 weeks_50 μM_rep2). (F) Correlation between representative samples from the 4 days and 4 weeks 50 μM exposure groups (4 days_50 μM_rep1 vs. 4 weeks_50 μM_rep1). Each point represents an individual transcript. Pearson correlation coefficients (R) and associated significance values are shown within each panel. Gene expression values were transformed prior to correlation analysis.

**Figure S3.** Benchmark dose (BMD) accumulation analyses for acute and chronic GenX exposure. Best benchmark dose lower confidence limit (BMDL) (A) and benchmark dose upper confidence limit (BMDU) (B) generated from transcriptomic benchmark dose modeling of GenX-exposed 3D HepG2/C3A spheroids. Curves represent the cumulative number of genes meeting benchmark dose modeling criteria across increasing BMD values. Red lines indicate acute exposure (4 days), and blue lines indicate chronic exposure (4 weeks).
